# A Ribonucleotide ↔ Phosphoramidate Reaction Network Optimized by Computer-Aided Design

**DOI:** 10.1021/jacs.2c05861

**Published:** 2022-08-11

**Authors:** Andreas Englert, Julian F. Vogel, Tim Bergner, Jessica Loske, Max von Delius

**Affiliations:** † Institute of Organic Chemistry, Ulm University, Albert-Einstein-Allee 11, 89081 Ulm, Germany; ‡ Central Facility for Electron Microscopy, Ulm University, Albert-Einstein-Allee 11, 89081 Ulm, Germany

## Abstract

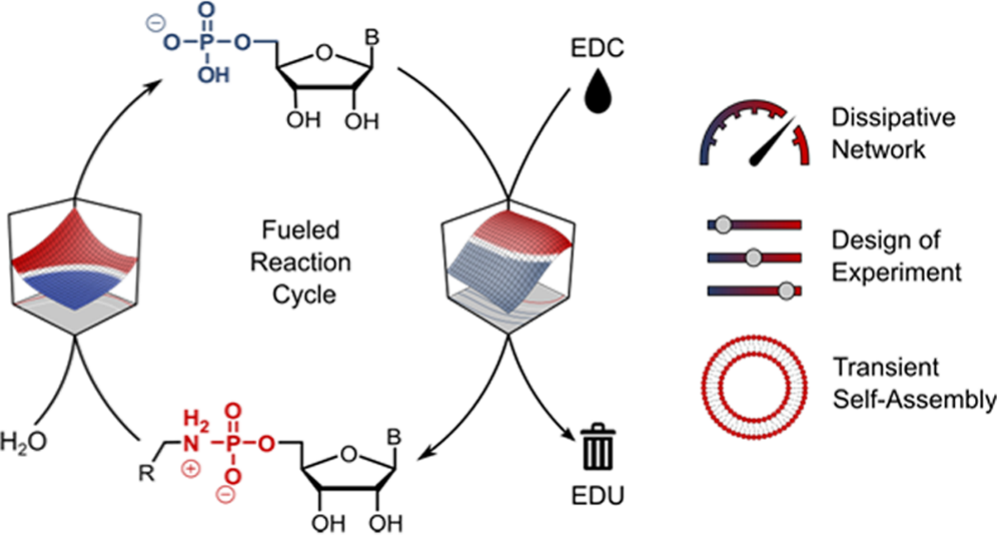

A growing number of out-of-equilibrium systems have been created and investigated in chemical laboratories over the past decade. One way to achieve this is to create a reaction cycle, in which the forward reaction is driven by a chemical fuel and the backward reaction follows a different pathway. Such dissipative reaction networks are still relatively rare, however, and most non-enzymatic examples are based on the carbodiimide-driven generation of carboxylic acid anhydrides. In this work, we describe a dissipative reaction network that comprises the chemically fueled formation of phosphoramidates from natural ribonucleotides (e.g., GMP or AMP) and phosphoramidate hydrolysis as a mild backward reaction. Because the individual reactions are subject to a multitude of interconnected parameters, the software-assisted tool “Design of Experiments” (DoE) was a great asset for optimizing and understanding the network. One notable insight was the stark effect of the nucleophilic catalyst 1-ethylimidazole (EtIm) on the hydrolysis rate, which is reminiscent of the action of the histidine group in phosphoramidase enzymes (e.g., HINT1). We were also able to use the reaction cycle to generate transient self-assemblies, which were characterized by dynamic light scattering (DLS), confocal microscopy (CLSM), and cryogenic transmission electron microscopy (cryo-TEM). Because these compartments are based on prebiotically plausible building blocks, our findings may have relevance for origin-of-life scenarios.

## Introduction

At the molecular level, life is an out-of-equilibrium state that is characterized by a multitude of interconnected networks, each exhibiting complex collective behavior.^1^ The emergent functions of such systems can only be maintained as long as energy is dissipated in the form of the consumption of high-energy molecules that stem from metabolic pathways.^2^ In some cases, most notably in actin filaments^3^ and microtubules,^4^ chemical fuels drive the self-assembly of organic building blocks into transient supramolecular scaffolds. Over the past decade, researchers in the field of systems chemistry^5−7^ have begun to mimic these natural processes, using artificial building blocks, chemical fuels,^8^ and catalysts.^9−13^

Because the most widespread fuels in nature are adenosine triphosphate (ATP) and guanosine triphosphate (GTP), it is not surprising that artificial dissipative self-assemblies (DSA) have been realized with these compounds.^14−19^ However, research on artificial systems is not limited to natural fuels. Pioneering work by Boekhoven, Eelkema, and van Esch^20,21^ for instance has been based on the alkylating agent dimethyl sulfate. Other fuels used to generate DSA include dithionate,^22^ perborate,^23^ cyclodextrin,^24^ acetic anhydride,^25^ and amino acids.^26^ Arguably, the most popular chemical fuels in artificial dissipative networks, especially in those that do not require enzyme catalysis, are carbodiimides such as 1-ethyl-3-(3-dimethylaminopropyl) carbodiimide (EDC).^27^ Although the prebiotic plausibility of carbodiimide fuels is questionable,^28−30^ EDC is valuable as a model reagent because of its water solubility, slow background reaction with water, and its exceptionally well-understood reaction profile.^31^ For these reasons, EDC has been successfully used in a large number of recent studies on transient behavior. Dynamic vesicles have been formed by EDC-fueled self-assembly^32^ and were shown to give rise to selective behavior due to phase separation.^33^ Comparable systems were shown to facilitate transient macrocyclization (including host–guest chemistry),^34,35^ time-controlled tuning of polymer properties,^36,37^ control over molecular emission,^38^ and fueled self-regulating hydrogels.^39−41^ In all cases, EDC is used to transform carboxylic acids into the corresponding anhydrides.

Furthermore, carbodiimides recently fueled the ratcheted and directional motion of molecular machines.^42,43^ Mimicking nature’s dissipative systems can be crucial to understanding the origins of life, which requires out-of-equilibrium systems featuring self-replication, metabolism, and compartmentalization.^23,44,45^ Recently, phosphoramidates have been identified as a compound class with prebiotic relevance. For example, efficient non-enzymatic primer extension of 3′-NP DNA^46,47^ and template-directed synthesis of 3N′-5P′-RNA^48^ have been reported (self-replication). Moreover, it was shown that prebiotically plausible stereoselective aminoacyl-RNA synthesis^28^ and even ribosome-like translation^49^ (metabolism) contain phosphoramidate intermediates. Both the formation^50−53^ and the hydrolysis^54−57^ as well as the self-assembly^58,59^ of phosphoramidates have been studied individually. However, a fueled reaction network based on phosphoramidates and DSA controlled by this chemistry is elusive.

**Figure 1:**
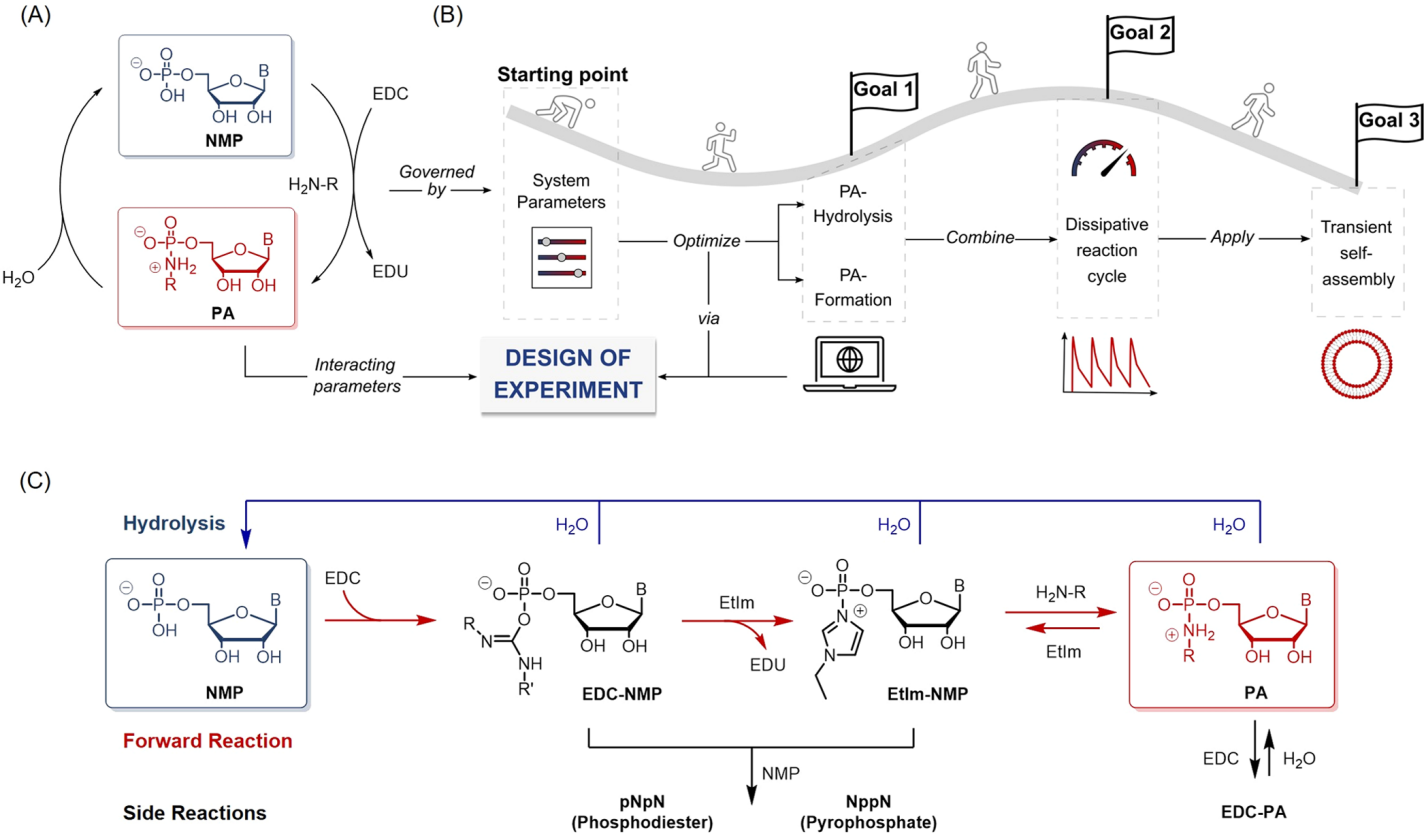
Reaction scheme and outline. (A) General scheme of the dissipative reaction cycle. (B) Objectives of this study and role of design of experiments (DoE). (C) Detailed reaction scheme of the reaction network, including the forward reaction from monophosphates NMP to phosphoramidates PA (red arrows), hydrolysis pathways (blue arrows), and side reactions (black arrows). B: nucleobase.

Herein, we present a dissipative reaction cycle, in which phosphoramidates are formed by the EDC-driven reaction of natural ribonucleotides with primary amines (Figure 1A). The hydrolytic backward reaction proceeds under relatively mild conditions, as long as it is catalyzed by 1-ethylimidazole (EtIm). Both the forward and the backward reaction were investigated by a broad design of experiments (DoE) approach (Figure 1B), which not only helped optimize the two fundamental processes but also revealed unexpected interactions between system parameters. Establishing ideal conditions for the full reaction cycle allowed us to demonstrate the transient self-assembly of rudimentary compartments comprising prebiotically plausible building blocks.

## Results and Discussion

### Phosphoramidate-Based Reaction Cycle

The reaction network comprises a three-step forward reaction from natural ribonucleotide monophosphate (NMP) to the corresponding phosphoramidate (PA, red arrows in Figure 1C). First, the phosphate group of the NMPs reacts with the chemical fuel 1-ethyl-3-(3-dimethylaminopropyl) carbodiimide (EDC) to form an activated phosphate (EDC-NMP). Richert and co-workers have shown that this intermediate is highly sensitive to hydrolysis, while 1-ethylimidazole (EtIm) is used as a catalyst to capture the EDC-NMP and form a more stable EtIm-activated phosphate (EtIm-NMP).^60^

Nucleophilic attack of a primary amine leads to the formation of a phosphoramidate. As we describe below in more details, the conversion of the PA back to the EtIm-NMP plays a crucial role in the (mild) hydrolysis of the PA, which is required to close the dissipative reaction cycle. Hydrolysis can occur for all intermediates along the forward reaction (blue arrows in Figure 1C). Additionally, some side reactions need to be taken into account (black arrows in Figure 1C). The activated phosphates can undergo a nucleophilic attack with the 2′- or 3′-hydroxy group of another ribose to form a phosphodiester (pNpN) or with a second 5′-phosphate to form a pyrophosphate (NppN). As previously reported by our group, pyrophosphates can form rapidly and are kinetically stable toward hydrolysis,^61^ which makes this side-reaction a problematic kinetic sink, while phosphodiesters hydrolyze more readily and played a negligible role in this study. Finally, we observed small amounts of *O*-acyl- and *N*-acyl-adducts (EDC-PA) between PA and EDC. The full chemical structures of these side products are depicted in Scheme S1. We refer to EDC as a “chemical fuel” to signify that the difference in free energy between EDC and the corresponding urea waste (EDU) is used to maintain a certain function,^8^ e.g., the generation of transient vesicles. In a proof-of-principle experiment, we show that continuous addition of the fuel leads to an out-of-equilibrium steady state, where fuel is dissipated over a long period of time (see the Supporting Information, Section VII).

The three main objectives of this work are illustrated in Figure 1B. We first aimed to investigate and optimize the PA hydrolysis and PA formation reactions individually. Parameter optimization for chemical reactions can be a time-consuming and difficult endeavor, especially when parameters are involved that interact with each other and thus cannot be considered independently. Classical “One Factor at a Time” (OFAT) strategies are not able to reveal such interactions and do not lead to efficient experimental designs.^62,63^ Particularly, in dissipative systems that possess two different reaction pathways, an OFAT approach might not be advisable. Computer-assisted design tools such as design of experiments (DoE) are well suited for the efficient evaluation of systems made up of interacting parameters and are applied in modern synthesis laboratories for the characterization and optimization of reactions.^64,65^ Furthermore, by conducting a sufficient number of runs, response surface methods (RSM) can be used within a DoE to establish a predictive description model. To the best of our knowledge, the use of DoE has not yet been reported in the context of dissipative reaction cycles.

Our second objective was to combine the findings of both DoEs to find a parameter set that satisfies all criteria to allow several reaction cycles upon chemical fueling. Finally, we aimed to use the reaction cycle for the dissipative self-assembly of prebiotically relevant compartments.

### Optimizing and Understanding the Hydrolysis of Phosphoramidates

**Figure 2:**
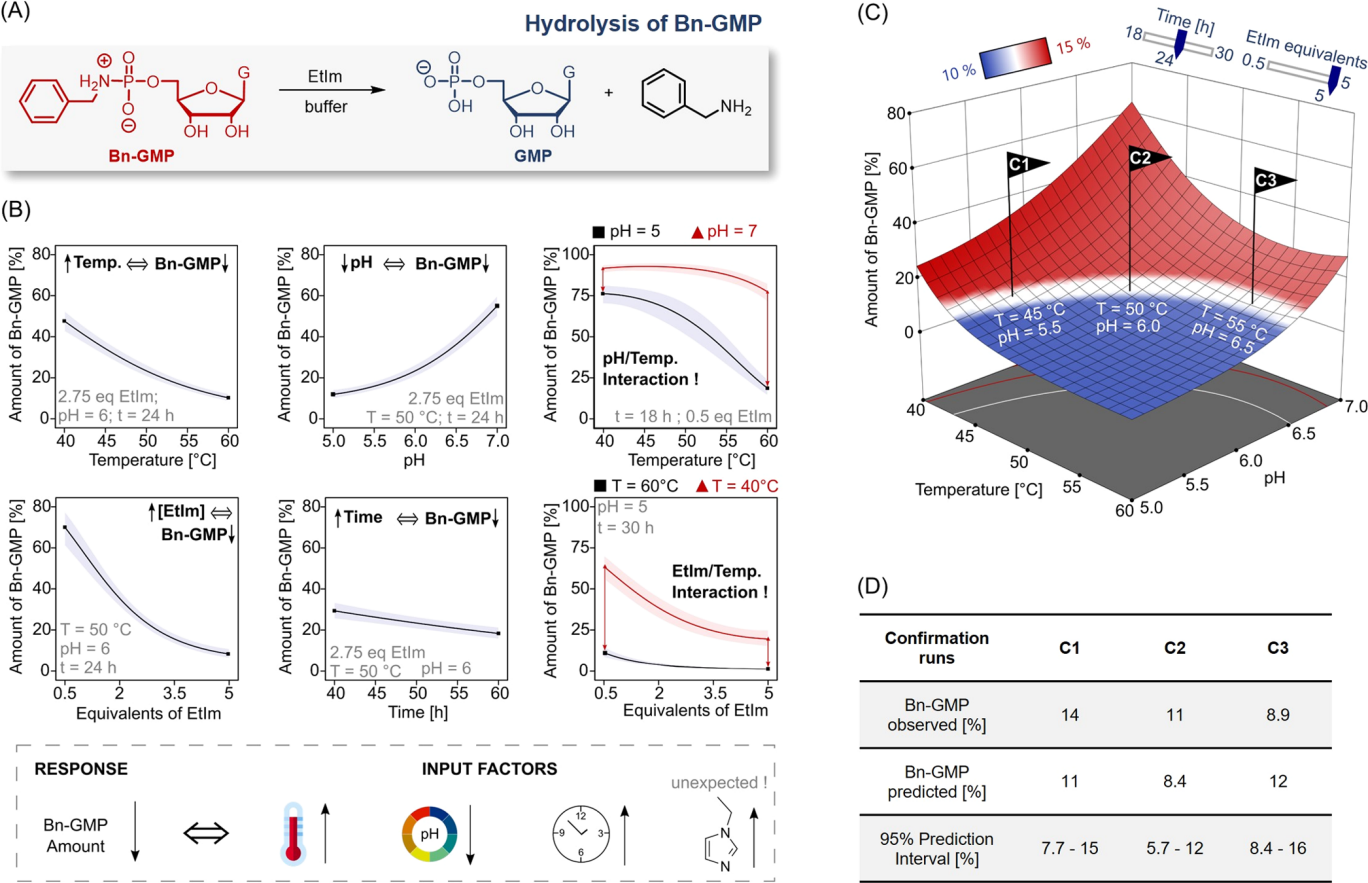
(A) Reaction scheme of Bn-GMP hydrolysis. (B) One-factor diagrams and interaction diagrams with 95% confidence intervals (shaded area). The constant parameter sets for each diagram are indicated. General trends observed for the hydrolysis reaction are summarized at the bottom. (C) Predicted response surface of the hydrolysis reaction. The flags C1, C2, and C3 indicate the experimental confirmation runs of the model. (D) Table of the confirmation runs, comparing the experimentally observed values with the response predicted by DoE.

To establish a dissipative reaction cycle, the backward reaction should proceed at a reasonable rate. We used the hydrolysis reaction depicted in Figure 2A as a reference system to evaluate whether phosphoramidates of primary amines fulfill this requirement. Bn-GMP was chosen as a model compound, and the respective reactions were performed at 25 mM concentration. The amount of Bn-GMP was determined by HPLC (with an internal standard) and was monitored throughout experiments to assess the degree of hydrolysis. To determine general trends and construct a predictive description model, we applied the DoE methodology to this system. More specifically, the reactions were performed in two blocks according to a central composite design (CCD) (Table S3), correlating the influence of the input factors temperature, pH, EtIm concentration, and time with the amount of unhydrolyzed Bn-GMP. The description model and the respective fit statistics are shown and discussed in detail in the Supporting Information (Section IV).

The observed trends are depicted schematically in Figure 2B. An increase in temperature, a more acidic pH, longer reaction times, and an increased amount of EtIm result in faster hydrolysis. The one-factor diagrams and interaction diagrams in Figure 2B show the quantitative effect of the individual factors on the response for different factor settings. While the correlation of reaction progress with temperature and time is unremarkable, the effect of pH is more interesting. In principle, we can explain this finding by the higher degree of protonation of the basic nitrogen atoms in the phosphoramidate and the phosphorimidazolide intermediate. This protonation is known to affect the rate of elimination of the leaving group in this “S_N_2-like” reaction.^55^ The pronounced influence of the EtIm amount on the hydrolysis was surprising, even though it had been reported previously that imidazole derivatives can play a role in the hydrolysis of phosphoramidates.^57^ EtIm as a hydrolysis catalyst offers a great advantage compared to other degradation reactions of dissipative reaction cycles since it serves as an additional parameter allowing one to fine-tune the reaction.

One benefit of DoE analysis is that factors are not treated independently, and it therefore accounts for the fact that adjusting one factor may be highly dependent on the setting of another factor. We observed this behavior clearly in the pH/temperature and EtIm/temperature interactions depicted in Figure 2B (right-hand side). The more the red and blue slopes in the interaction diagrams differ from each other, the stronger the interaction. For instance, increasing the pH at 60 °C leads to starkly increased hydrolysis when compared to the moderate increase observed at 40 °C. Because such interactions are likely to exist also in other reaction networks, we believe the DoE methodology can generally be applied to help identify suitable reaction conditions.

We chose DoE based on RSM designs for the analysis of the system because this approach not only reveals trends within the recorded data set but also allows predictions based on the description model. This predictive capacity is demonstrated in the temperature-pH-response surface shown in Figure 2C. As mentioned above, the goal of this DoE was to achieve nearly complete hydrolysis of the reference compound within a reasonable timeframe. Therefore, three confirmation runs (C1–C3) were performed under conditions where a Bn-GMP amount below 15% after 24 h was predicted by the model. The mean values of the experimentally observed Bn-GMP amount of three parallel measurements are depicted in Figure 2D. All three confirmation runs are in good agreement with the predictions and within the interval of the prediction error, thus corroborating that we can predict diverse conditions for reasonably fast hydrolysis.

### Optimizing and Understanding the EDC-Driven Formation of Phosphoramidates

**Figure 3:**
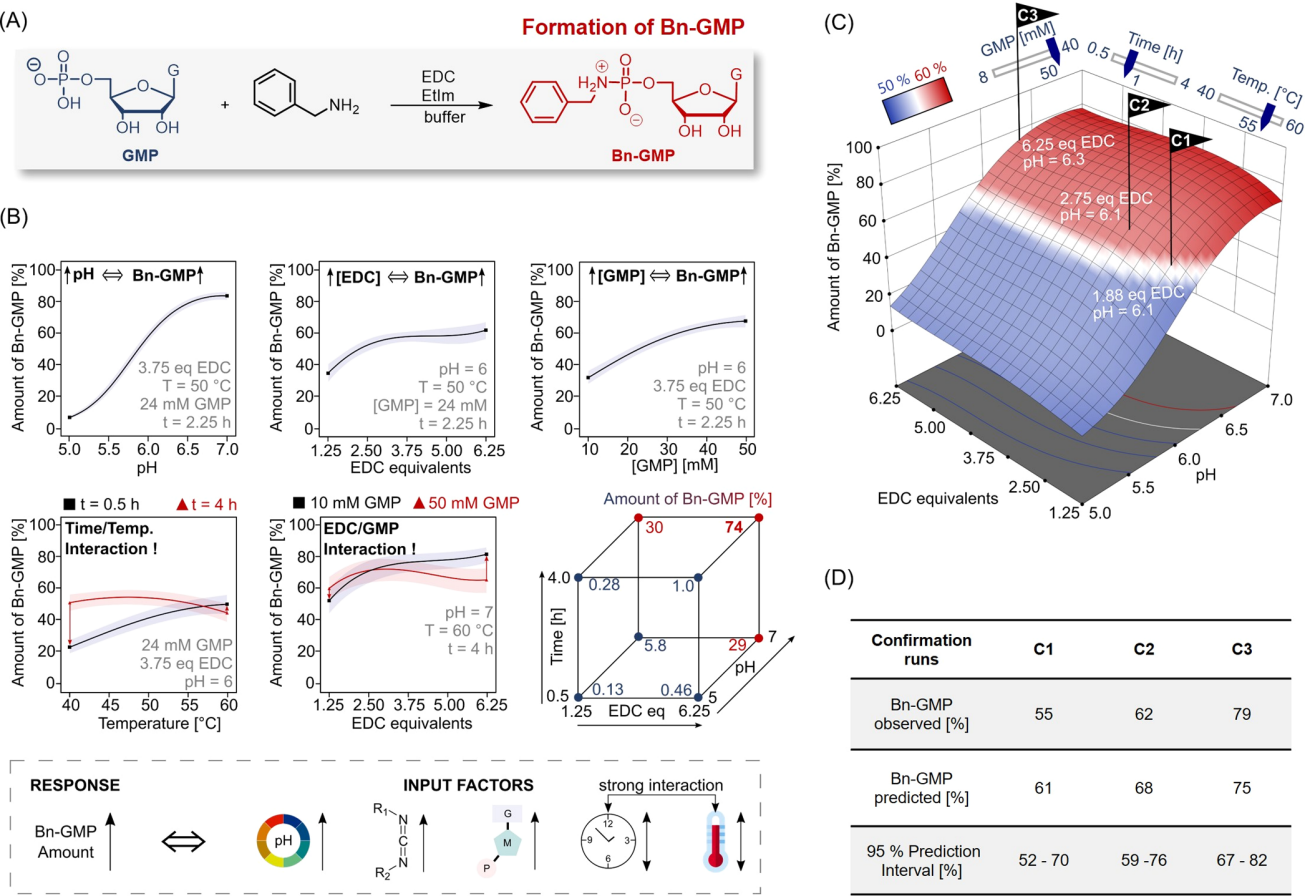
(A) Reaction scheme of Bn-GMP formation. (B) One-factor diagrams and interaction diagrams with 95% confidence intervals (shaded area). All experiments were performed containing five equivalents of EtIm. Further parameter sets held constant are stated for each diagram. In the full factorial representation (cube), the data given here correspond to a temperature of 40 °C and an 8 mM GMP concentration. The general trends of the PA formation reaction are summarized at the bottom. (C) Predicted response surface of the formation reaction. The flags C1, C2, and C3 indicate the experimental confirmation runs of the model. (D) Table of the confirmation runs, comparing the experimentally observed values with the response predicted by DoE.

After evaluating the hydrolysis of phosphoramidates (backward reaction), we turned our attention to the forward reaction. To ensure comparability, we used the same model compounds as above, and the only crucial difference from the hydrolysis reaction is the presence of EDC (Figure 3A). Again, the amount of Bn-GMP was determined by HPLC and used as a response to evaluate the efficiency of the reaction. This response was correlated with the five input factors pH, temperature, concentration of GMP, time, and equivalents of EDC. The factor settings, the experimental design, and the respective fit statistics of the description model are shown in the Supporting Information (Section V). As shown in Figure 3B, increasing the pH, equivalents of EDC, and the concentration of GMP results in a more efficient Bn-GMP formation. The impact of these factors is illustrated in Figure 3B in the one-factor and interaction diagrams. Especially, the pH turned out to influence the reaction tremendously (steep curve in Figure 3B, top left). Under acidic conditions, the protonated state of the amine is favored, which impedes its effective nucleophilicity. Additionally, as the hydrolysis of Bn-GMP proceeds faster at a lower pH, it is reasonable to assume that the reactive intermediates (EDC-GMP and EtIm-GMP) also hydrolyze faster at a lower pH. The influence of EDC and GMP can be explained accordingly. Increasing the amount of the activation agent EDC counterbalances the hydrolysis of reactive intermediates, while a higher GMP concentration naturally increases the forward rate (according to collision theory).

In the case of the input factors temperature and time, no general trend could be established, as shown in the first interaction diagram of Figure 3B (for further time interactions, see Figure S8). The inverse slopes of the two graphs indicate that high Bn-GMP yields are observed after long reaction times at relatively low temperatures, while high yields can also be observed after short reaction times at relatively high temperatures. Such a behavior is characteristic of dissipative reaction cycles as the backward reaction dominates earlier at high temperatures. Moreover, we found that side reactions are also capable of diminishing the yield of the reaction, as illustrated in the second interaction diagram (Figure 3B).

Even though EDC reacts preferentially with the phosphate, a high excess of this reagent leads to the formation of EDC adducts (as shown in the reaction scheme in Figure 1C) and therefore an otherwise unexplainable decrease in Bn-GMP formation. The importance of multifactor interactions is further highlighted by the cubic representation of the parameters time, EDC, and pH (cube in Figure 3B).^66^ Only careful adjustment of all three parameters allowed a considerable increase of the reaction efficiency. Figure 3C shows the EDC-pH-response surface of the investigated reaction as predicted by DoE. As with the hydrolysis reaction, confirmation runs (C1–C3) were conducted at conditions where the reaction yields were predicted to be above 50%. All three confirmation runs agree with the predictions and are within the interval of the prediction error (Figure 3D).

### Closing the Dissipative Reaction Cycle

**Figure 4:**
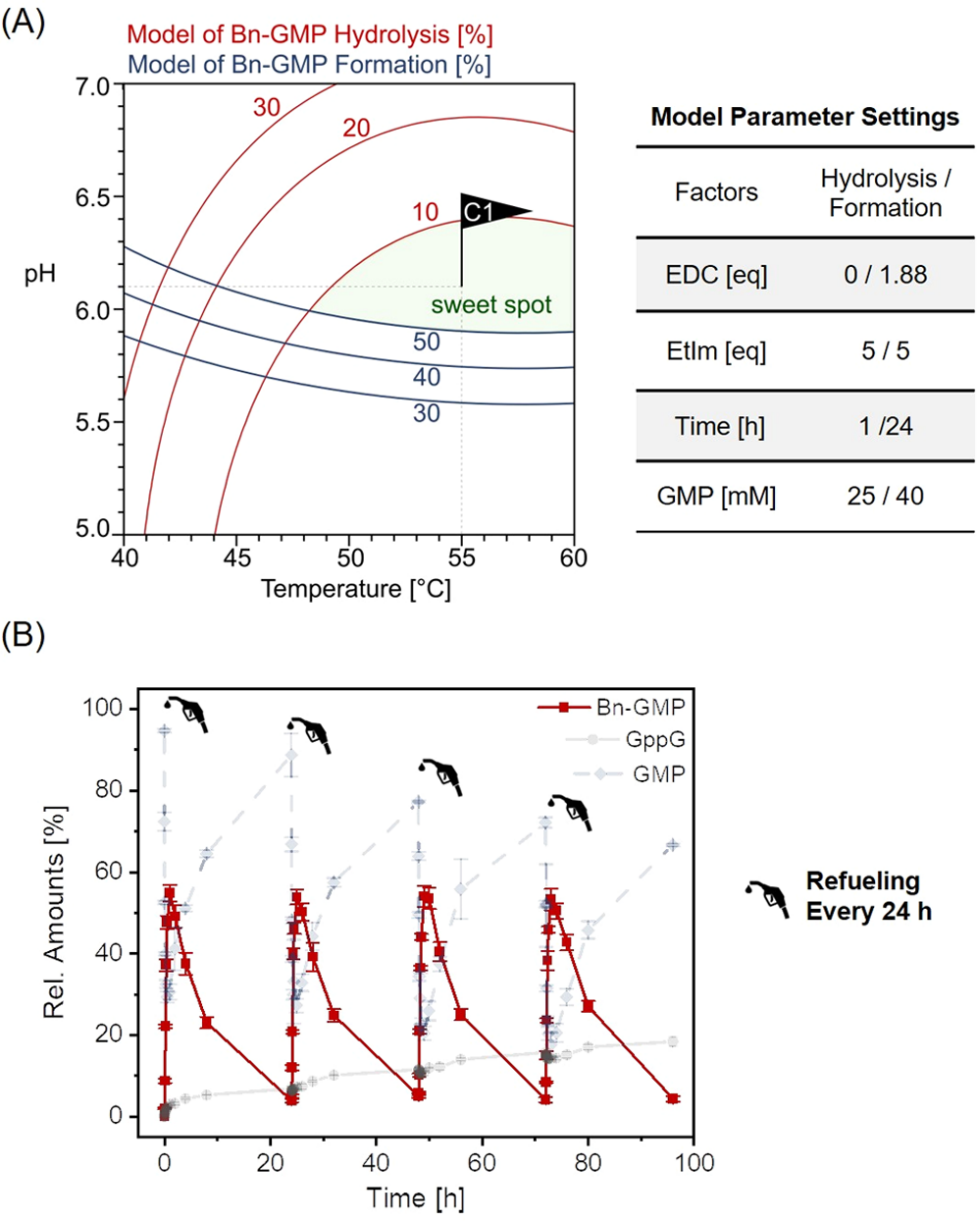
Combination of PA hydrolysis and PA formation (DoE). (A) Graphical optimization of temperature and pH for a dissipative reaction cycle. The desired conditions allow for hydrolysis below 10% of the residual Bn-GMP within 24 h (red lines) and sufficient (over 50%) Bn-GMP formation (blue lines) at the same time. The table specifies the remaining system parameters. (B) Four dissipative reaction cycles under optimized conditions. Relative amounts of Bn-GMP (red), GMP (blue, dotted line), and GppG (light gray) as determined by HPLC. Error bars indicate standard deviation based on triplicate reaction runs. Data points are connected to guide the eye.

By conducting the DoEs, we found general system trends and description models. With these results in hand, our next objective was to use the reference system to establish a dissipative reaction cycle by combining the above findings. Figure 4A shows a contour plot that combines both models and was used to identify conditions suited for an efficient dissipative reaction cycle. Specifically, the green-shaded area represents conditions under which the PA hydrolysis occurs nearly completely within 24 h, but the PA formation is still quite effective (yield higher than 50%). To test the validity of the model, we fueled the dissipative system four times with EDC under these conditions (55 °C, pH = 6.1; see Table in Figure 4A). Figure 4B shows the relative amount of Bn-GMP over four cycles, validating that the predicted conditions are suited to establish a dissipative reaction cycle. It must be noted, however, that side products such as the pyrophosphate GppG form and accumulate over the course of several reaction cycles. For applications targeting many cycles in dissipative systems, the exploration of catalysts that enable the hydrolysis of pyrophosphates may be necessary.^61^

### Transient Self-Assembly

**Figure 5:**
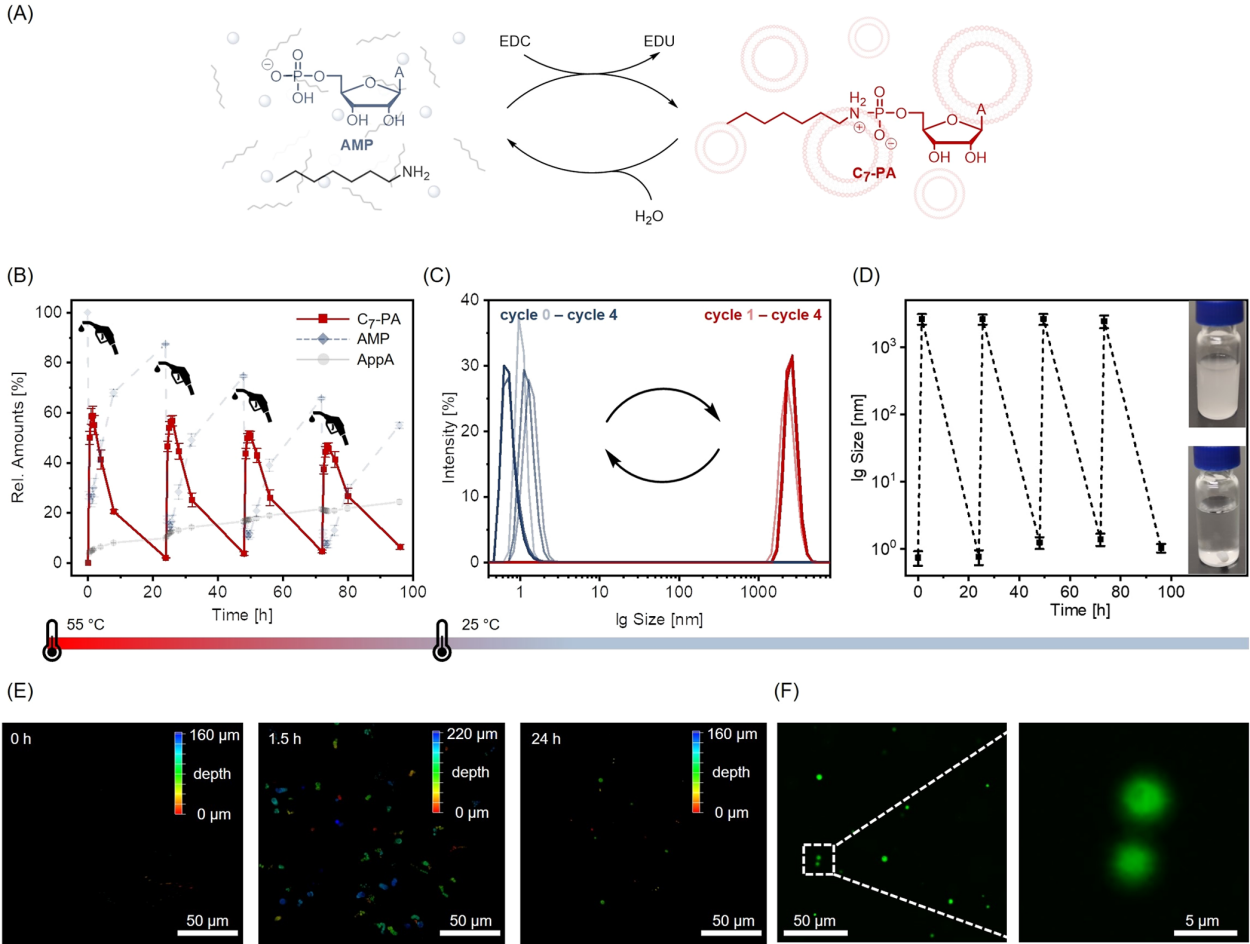
Transient self-assembly of compartments. (A) Reaction scheme of the EDC-driven reaction cycle. (B) Four dissipative reaction cycles (performed at 55 °C). Relative amounts of C_7_-PA (red), AMP (blue, dotted line), and AppA (light gray) as determined by HPLC. Error bars indicate standard deviation based on triplicate reaction runs. Data points are connected to guide the eye. (C) DLS measurements at different time points during the four cycles (measured at 25 °C). The average distributions in mean hydrodynamic diameter of five parallel measurements are shown. (D) Average size of the aggregates at different time points as measured by DLS. Error bars indicate one standard deviation. Dotted lines are displayed to guide the eye. Inlets show clear solutions in the non-aggregated and turbid solutions in the aggregated state, respectively (at 25 °C). (E) 3D-CLSM images during one reaction cycle, measured at 25 °C and along the whole *z*-axis. The color code indicates the depth of the aggregates in the sample. (F) 2D-CLSM image of vesicular structures after 1.5 h with magnification. As a fluorescent dye 2.5 μM C153 was used.

Having optimized the dissipative reaction cycle, we wondered whether the combination of an alkylamine with a monophosphate could give rise to the transient self-assembly of compartments (Figure 5A). Due to the simplicity of all building blocks (except perhaps the fuel), such a system may have prebiotic relevance.^28,47,49^ Furthermore, the use of an alkylamine rather than benzylamine demonstrates the relatively broad scope of the approach. Using alkylamines required only moderate adjustment of the reaction conditions, probably due to differences in p*K*_a_.^67^ We dissolved the natural ribonucleotide adenosine 5’-monophosphate (AMP) (75 mM) in a buffer, containing 5 equiv EtIm and added 1.5 equiv heptylamine. After adjusting the pH to 6.5 (at 55 °C) we obtained a clear, molecularly dissolved solution. Addition of 2 equiv EDC led to the rapid formation of the corresponding phosphoramidate (C_7_-PA), as determined by quantitative HPLC (Figures 5B and S12). After 1.5 h, 60% of the AMP was converted to C_7_-PA. As soon as the fuel was depleted, C_7_-PA began to hydrolyze, and its amount dropped below 10% within 24 h. These results imply that the “sweet spot” identified in Figure 4A for benzyl amine is also viable for alkyl amines with only minor adjustments, and the stage was therefore set for the coupling of dissipative cycles with hydrophobic self-assembly. As shown in Figure 5B, we were able to demonstrate that this reaction cycle can be (re-)fueled at least four times. The main side-reaction is the formation of adenosine pyrophosphate (AppA). While the reaction was performed at 55 °C, we were only able to observe transient self-assembly at lower temperatures, e.g., at 25 °C. When quickly cooling the reaction mixture to 25 °C after 1.5 h, we obtained a turbid solution, and dynamic light scattering (DLS) revealed the formation of aggregates with an average hydrodynamic diameter of around 2.6 μm (Figures 5C,D and S13). After 24 h, the solutions were clear at 25 °C, and DLS indicated that the aggregates had vanished upon hydrolysis. Figure 5C shows the hydrodynamic diameter as an average of five measurements, corroborating the good reversibility of the self-assembly during four reaction cycles. Finally, confocal laser scanning microscopy (CLSM) with 2.5 μM “Cumarin 153” (C153) as a fluorescent dye was used to directly visualize the transient formation of aggregates (Figures 5E,F and S14). We scanned the sample along the whole z-axis to obtain a three-dimensional image of the solution (the color code in Figure 5E indicates the depth of the vesicle along the *z*-axis). While we initially observed no aggregates, a large number of spherical objects with diameters of approx 1–5 μm were visible 1.5 h after the addition of EDC. After 24 h, when most of the C_7_-PA had hydrolyzed, there were only very few aggregates left. Figure 5F shows an enlarged two-dimensional CLSM image (after 1.5 h) of two aggregates, which would be consistent with a vesicular or colloidal structure. Cryogenic transmission electron microscopy (cryo-TEM, Figures S15 and S16) is rather indicative of vesicles. Irrespective of the aggregate structure, the DLS and CLSM measurements reveal how the phosphoramidate-based reaction cycle we introduce herein can lead to compartmentalization under primordial conditions with prebiotically plausible building blocks such as ribonucleotides^68^ and primary amines.^69^ Our finding that the activation chemistry proceeded most efficiently at 55 °C, whereas compartments were only present at 25 °C, is interesting in the context of studies on thermophoresis, which showed that temperature gradients (e.g., at hydrothermal vents)^70^ can lead to the enrichment of prebiotic molecules^71^ and large oligomers^72^ as well as the fission of lipid vesicles that can serve as a model for protocells.^73^ Despite the use of EDC as a model reagent, our work therefore suggests that temperature gradients could have prebiotic relevance not only for physical or simple chemical processes but also for the regulation of individual processes in chemical reaction cycles and for the dynamic kinetic stability of dissipative compartments.

## Conclusions

We report an out-of-equilibrium reaction cycle based on natural ribonucleotides and their corresponding phosphoramidates. For the first time, the software-assisted tool design of experiments (DoE) was used to optimize and better understand a fuel-driven chemical reaction cycle. For instance, we discovered a strong influence of 1-ethylimidazole on the hydrolysis of phosphoramidates. Such catalysis is reminiscent of the action of the histidine group in phosphoramidase enzymes (e.g., HINT1)^74^ and has been used strategically before by others.^25,60^ In the case of the phosphoramidate formation, we identified the pH value as the most important parameter and revealed interesting time/temperature interactions that are characteristic of dissipative systems. Furthermore, we were able to identify a “sweet spot” set of reaction conditions for both the forward and the backward reaction to enable an efficient dissipative reaction cycle. In all cases, we found the modeled predictions to be in good agreement with our experimental results. We expect that the DoE approach will become more commonplace in systems chemistry due to its ability to reveal useful insights into chemical systems governed by a complex set of parameters.

We also designed and realized a system capable of undergoing transient self-assembly. While both self-replicating and metabolic phosphoramidate-based systems have been reported recently,^28,47,49^ to the best of our knowledge, we report the first example of transient compartmentalization using this particular chemistry. Despite some limitations related to the fuel (EDC) and the accumulation of a side product (pyrophosphate), this work therefore underscores the potential importance of phosphoramidates in prebiotic chemistry.

## Supporting Information Available

Reaction conditions, DoE data (such as factor settings, experimental design, and respective fit statistics of the description model), and additional measurements such as HPLC, DLS, CLSM, and (cryo-)TEM (PDF)
Raw_Data (XLSX)

## References

[ref1] Ruiz-Mirazo K.; Briones C.; De La Escosura A. Prebiotic Systems Chemistry: New Perspectives for the Origins of Life. Chem. Rev. 2014, 114, 285–366. 10.1021/cr2004844. 24171674

[ref2] Giuseppone N.; Walther A. Out-of-Equilibrium (Supra)Molecular Systems and Materials, Wiley-VCH, 2021; pp 167–169 10.1002/9783527821990.

[ref3] Pollard T. D.; Blanchoin L.; Mullins R. D. Molecular Mechanism Controlling Actin Filament Dynamics in Nonmuscle Cells. Annu. Rev. Biophys. Biomol. Struct. 2000, 29, 545–576. 10.1146/annurev.biophys.29.1.545. 10940259

[ref4] Conde C.; Cáceres A. Microtubule Assembly, Organization and Dynamics in Axons and Dendrites. Nat. Rev. Neurosci. 2009, 10, 319–332. 10.1038/nrn2631. 19377501

[ref5] Ashkenasy G.; Hermans T. M.; Otto S.; Taylor A. F. Systems Chemistry. Chem. Soc. Rev. 2017, 46, 2543–2554. 10.1039/C7CS00117G. 28418049

[ref6] Mattia E.; Otto S. Supramolecular Systems Chemistry. Nat. Nanotechnol. 2015, 10, 111–119. 10.1038/nnano.2014.337. 25652169

[ref7] Miljanić O. S. Small-Molecule Systems Chemistry. Chem 2017, 2, 502–524. 10.1016/j.chempr.2017.03.002.

[ref8] Borsley S.; Leigh D. A.; Roberts B. M. W. Chemical Fuels for Molecular Machinery. Nat. Chem. 2022, 14, 728–738. 10.1038/s41557-022-00970-9. 35778564

[ref9] Das K.; Gabrielli L.; Prins L. J. Chemically Fueled Self-Assembly in Biology and Chemistry. Angew. Chem., Int. Ed. 2021, 60, 20120–20143. 10.1002/anie.202100274. PMC8453758 33704885

[ref10] Rieß B.; Grötsch R. K.; Boekhoven J. The Design of Dissipative Molecular Assemblies Driven by Chemical Reaction Cycles. Chem 2020, 6, 552–578. 10.1016/j.chempr.2019.11.008.

[ref11] Wang G.; Liu S. Strategies to Construct a Chemical-Fuel-Driven Self-Assembly. ChemSystemsChem 2020, 2, e1900046 10.1002/syst.201900046.

[ref12] De S.; Klajn R. Dissipative Self-Assembly Driven by the Consumption of Chemical Fuels. Adv. Mater. 2018, 30, 1706750 10.1002/adma.201706750. 29520846

[ref13] Weißenfels M.; Gemen J.; Klajn R. Perspective Dissipative Self-Assembly: Fueling with Chemicals versus Light. Chem 2021, 7, 23–37. 10.1016/j.chempr.2020.11.025.

[ref14] Heinen L.; Walther A. Programmable Dynamic Steady States in ATP-Driven Nonequilibrium DNA Systems. Sci. Adv. 2019, 5, eaaw059 10.1126/sciadv.aaw0590. PMC6641946 31334349

[ref15] Deng J.; Walther A. ATP-Responsive and ATP-Fueled Self-Assembling Systems and Materials. Adv. Mater. 2020, 32, 2002629 10.1002/adma.202002629. 32881127

[ref16] Sorrenti A.; Leira-Iglesias J.; Sato A.; Hermans T. M. Non-Equilibrium Steady States in Supramolecular Polymerization. Nat. Commun. 2017, 8, 15899 10.1038/ncomms15899. 28627512 PMC5481825

[ref17] Maiti S.; Fortunati I.; Ferrante C.; Scrimin P.; Prins L. J. Dissipative Self-Assembly of Vesicular Nanoreactors. Nat. Chem. 2016, 8, 725–731. 10.1038/nchem.2511. 27325101

[ref18] Cardona M. A.; Prins L. J. ATP-Fuelled Self-Assembly to Regulate Chemical Reactivity in the Time Domain. Chem. Sci. 2020, 11, 1518–1522. 10.1039/C9SC05188K. PMC8148039 34084381

[ref19] Chen R.; Neri S.; Prins L. J. Enhanced Catalytic Activity under Non-Equilibrium Conditions. Nat. Nanotechnol. 2020, 15, 868–874. 10.1038/s41565-020-0734-1. 32690887

[ref20] Boekhoven J.; Brizard A. M.; Kowlgi K. N. K.; Koper G. J. M.; Eelkema R.; Van Esch J. H. Dissipative Self-Assembly of a Molecular Gelator by Using a Chemical Fuel. Angew. Chem., Int. Ed. 2010, 49, 4825–4828. 10.1002/anie.201001511. 20512834

[ref21] Boekhoven J.; Hendriksen W. E.; Koper G. J. M.; Eelkema R.; Esch J. H. Van. Transient Assembly of Active Materials Fueled by a Chemical Reaction. Science 2015, 349, 1075–1080. 10.1126/science.aac6103. 26339025

[ref22] Singh N.; Lainer B.; Formon G. J. M.; Piccoli S.; De; Hermans T. M. Re-Programming Hydrogel Properties Using a Fuel-Driven Reaction Cycle. J. Am. Chem. Soc. 2020, 142, 4083–4087. 10.1021/jacs.9b11503. 32065526

[ref23] Yang S.; Schaeffer G.; Mattia E.; Markovitch O.; Liu K.; Hussain A. S.; Ottelé J.; Sood A.; Otto S. Chemical Fueling Molecular Complexification of Self-Replicators. Angew. Chem., Int. Ed. 2021, 60, 1344–11349. 10.1002/anie.202185261. PMC8251556 33689197

[ref24] Lu H.; Hao J.; Wang X. Host-Fueled Transient Supramolecular Hydrogels. ChemSystemsChem 2022, 4, e202100050 10.1002/syst.202100050.

[ref25] Helm M. P.; Wang C. L.; Fan B.; Macchione M.; Mendes E.; Eelkema R. Organocatalytic Control over a Fuel-Driven Transient-Esterification Network. Angew. Chem., Int. Ed. 2020, 59, 20604–20611. 10.1002/anie.202008921. PMC7693295 32700406

[ref26] Debnath S.; Roy S.; Ulijn R. V. Peptide Nanofibers with Dynamic Instability through Nonequilibrium Biocatalytic Assembly. J. Am. Chem. Soc. 2013, 135, 16789–16792. 10.1021/ja4086353. 24147566

[ref27] Schwarz P. S.; Tena-solsona M.; Dai K.; Boekhoven J. Carbodiimide-Fueled Catalytic Reaction Cycles to Regulate Supramolecular Processes. Chem. Commun. 2022, 58, 1284–1297. 10.1039/D1CC06428B. 35014639

[ref28] Roberts S. J.; Liu Z.; Sutherland J. D. Potentially Prebiotic Synthesis of Aminoacyl-RNA via a Bridging Phosphoramidate-Ester Intermediate. J. Am. Chem. Soc. 2022, 144, 4254–4259. 10.1021/jacs.2c00772. 35230111 PMC9097472

[ref29] Song E. Y.; Jiménez E. I.; Lin H.; Le Vay K.; Krishnamurthy R.; Mutschler H. Prebiotically Plausible RNA Activation Compatible with Ribozyme-Catalyzed Ligation. Angew. Chem., Int. Ed. 2021, 60, 2952–2957. 10.1002/anie.202010918. PMC7898671 33128282

[ref30] Duvernay F.; Chiavassa T.; Borget F.; Aycard J. P. Experimental Study of Water-Ice Catalyzed Thermal Isomerization of Cyanamide into Carbodiimide: Implication for Prebiotic Chemistry. J. Am. Chem. Soc. 2004, 126, 7772–7773. 10.1021/ja048721b. 15212513

[ref31] Williams A.; Ibrahim I. T. Carbodiimide Chemistry: Recent Advances. Chem. Rev. 1981, 81, 589–636. 10.1021/cr00046a004.

[ref32] Wanzke C.; Jussupow A.; Kohler F.; Dietz H.; Kaila V. R. I.; Boekhoven J. Dynamic Vesicles Formed by Dissipative Self-Assembly. ChemSystemsChem 2020, 2, e190004 10.1002/syst.201900044.

[ref33] Tena-Solsona M.; Wanzke C.; Riess B.; Bausch A. R.; Boekhoven J. Self-Selection of Dissipative Assemblies Driven by Primitive Chemical Reaction Networks. Nat. Commun. 2018, 9, 2044 10.1038/s41467-018-04488-y. 29795292 PMC5966463

[ref34] Kariyawasam L. S.; Hartley C. S. Dissipative Assembly of Aqueous Carboxylic Acid Anhydrides Fueled by Carbodiimides. J. Am. Chem. Soc. 2017, 139, 11949–11955. 10.1021/jacs.7b06099. 28777554

[ref35] Hossain M. M.; Atkinson J. L.; Hartley C. S. Dissipative Assembly of Macrocycles Comprising Multiple Transient Bonds. Angew. Chem., Int. Ed. 2020, 59, 13807–13813. 10.1002/anie.202001523. 32384209

[ref36] Zhang B.; Jayalath I. M.; Ke J.; Sparks J. L.; Hartley C. S.; Konkolewicz D. Chemically Fueled Covalent Crosslinking of Polymer Materials. Chem. Commun. 2019, 55, 2086–2089. 10.1039/C8CC09823A. 30694271

[ref37] Würbser M. A.; Schwarz P. S.; Heckel J.; Bergmann A. M.; Walther A.; Boekhoven J. Chemically Fueled Block Copolymer Self-Assembly into Transient Nanoreactors. ChemSystemsChem 2021, 3, e2100015 10.1002/syst.202100015.

[ref38] Zong Z.; Zhang Q.; Qiu S. H.; Wang Q.; Zhao C.; Zhao C. X.; Tian H.; Qu D. H. Dynamic Timing Control over Multicolor Molecular Emission by Temporal Chemical Locking. Angew. Chem., Int. Ed. 2022, 61, e2021164 10.1002/anie.202116414. 35072333

[ref39] Bal S.; Das K.; Ahmed S.; Das D. Chemically Fueled Dissipative Self-Assembly That Exploits Cooperative Catalysis. Angew. Chem., Int. Ed. 2019, 58, 244–247. 10.1002/anie.201811749. 30395376

[ref40] Panja S.; Dietrich B.; Adams D. J. Chemically Fuelled Self-Regulating Gel-to-Gel Transition. ChemSystemsChem 2019, 1900038, 2–7. 10.1002/syst.201900038.

[ref41] Heckel J.; Loescher S.; Mathers R. T.; Walther A. Chemically Fueled Volume Phase Transition of Polyacid Microgels. Angew. Chem., Int. Ed. 2021, 60, 7117–7125. 10.1002/anie.202014417. PMC8048534 33340387

[ref42] Borsley S.; Leigh D. A.; Roberts B. M. W. A Doubly Kinetically-Gated Information Ratchet Autonomously Driven by Carbodiimide Hydration. J. Am. Chem. Soc. 2021, 143, 4414–4420. 10.1021/jacs.1c01172. 33705123

[ref43] Borsley S.; Kreidt E.; Leigh D. A.; Roberts B. M. W. Autonomous Fuelled Directional Rotation about a Covalent Single Bond. Nature 2022, 604, 80–85. 10.1038/s41586-022-04450-5. 35388198

[ref44] Adamski P.; Eleveld M.; Sood A.; Kun Á.; Szilágyi A.; Czárán T.; Szathmáry E.; Otto S. From Self-Replication to Replicator Systems En Route to de Novo Life. Nat. Rev. 2020, 4, 386–403. 10.1038/s41570-020-0196-x. 37127968

[ref45] Otto S. An Approach to the De Novo Synthesis of Life. Acc. Chem. Res. 2022, 55, 145–155. 10.1021/acs.accounts.1c00534. 34964346 PMC8772268

[ref46] Zhang S.; Zhang N.; Blain J. C.; Szostak J. W. Synthesis of N3′-P5′-Linked Phosphoramidate DNA by Nonenzymatic Template-Directed Primer Extension. J. Am. Chem. Soc. 2013, 135, 924–932. 10.1021/ja311164j. 23252395 PMC3548433

[ref47] Zhou L.; Szostak J. W. Nonenzymatic Template-Directed Synthesis of Mixed-Sequence 3′-NP-DNA up to 25 Nucleotides Long Inside Model Protocells. J. Am. Chem. Soc. 2019, 141, 10481–10488. 10.1021/jacs.9b04858. 31180644 PMC7547854

[ref48] Kaiser A.; Spies S.; Lommel T.; Richert C. Template-Directed Synthesis in 3′- and 5′-Direction with Reversible Termination. Angew. Chem., Int. Ed. 2012, 51, 8299–8303. 10.1093/nar/gkq1293. 22777755

[ref49] Jash B.; Tremmel P.; Jovanovic D.; Richert C. Single Nucleotide Translation without Ribosomes. Nat. Chem. 2021, 13, 751–757. 10.1038/s41557-021-00749-4. 34312504

[ref50] Gryaznov S. M.; Lloyd D. H.; Chen J.; Schultz R. G.; Dedionisio L. A.; Ratmeyert L.; Wilson W. D. Oligonucleotide N3′-P5′ Phosphoramidates. Proc. Natl. Acad. Sci. U.S.A. 1995, 92, 5798–5802. 7541136 10.1073/pnas.92.13.5798 PMC41588

[ref51] Chen J.-K.; Schultz R. G.; Lioyd D. H.; Gryaznov S. M. Synthesis of Oligodeoxyribonucleotide N3′→P5′ Phosphoramidates. Nucleic Acids Res. 1995, 23, 2661–2668. 10.1093/nar/23.14.2661. 7651827 PMC307090

[ref52] Griesser H.; Tremmel P.; Kervio E.; Pfeffer C.; Steiner U. E.; Richert C. Ribonucleotides and RNA Promote Peptide Chain Growth. Angew. Chem., Int. Ed. 2017, 56, 1219–1223. 10.1002/anie.201610650. 28000995

[ref53] Griesser H.; Bechthold M.; Tremmel P.; Kervio E.; Richert C. Amino Acid-Specific, Ribonucleotide-Promoted Peptide Formation in the Absence of Enzymes. Angew. Chem., Int. Ed. 2017, 56, 1224–1228. 10.1002/anie.201610651. 28000974

[ref54] Lonnberg T.; Ora M.; Lonnberg H. Hydrolytic Reactions of Nucleoside Phosphoramidates: Kinetics and Mechanisms. Org. Chem. 2010, 7, 33–43.

[ref55] Maiti M.; Michielssens S.; Dyubankova N.; Maiti M.; Lescrinier E.; Ceulemans A.; Herdewijn P. Influence of the Nucleobase and Anchimeric Assistance of the Carboxyl Acid Groups in the Hydrolysis of Amino Acid Nucleoside Phosphoramidates. Chem. - Eur. J. 2012, 18, 857–868. 10.1002/chem.201102279. 22173724

[ref56] Choy C. J.; Ley C. R.; Davis A. L.; Backer B. S.; Geruntho J. J.; Clowers B. H.; Berkman C. E. Second-Generation Tunable PH-Sensitive Phosphoramidate-Based Linkers for Controlled Release. Bioconjugate Chem. 2016, 27, 2206–2213. 10.1021/acs.bioconjchem.6b00422. 27562353

[ref57] Jovanovic D.; Tremmel P.; Pallan P. S.; Egli M.; Richert C. The Enzyme-Free Release of Nucleotides from Phosphoramidates Depends Strongly on the Amino Acid. Angew. Chem., Int. Ed. 2020, 59, 20154–20160. 10.1002/anie.202008665. PMC7436718 32757352

[ref58] Gangadhara K. L.; Srivastava P.; Rozenski J.; Mattelaer H. P.; Leen V.; Dehaen W.; Hofkens J.; Lescrinier E.; Herdewijn P. Design and Synthesis of Nucleolipids as Possible Activated Precursors for Oligomer Formation via Intramolecular Catalysis: Stability Study and Supramolecular Organization. J. Syst. Chem. 2014, 5, 5 10.1186/s13322-014-0005-3. 25558290 PMC4279058

[ref59] West H. T.; Csizmar C. M.; Wagner C. R. Tunable Supramolecular Assemblies from Amphiphilic Nucleoside Phosphoramidate Nanofibers by Enzyme Activation. Biomacromolecules 2018, 19, 2650–2656. 10.1021/acs.biomac.8b00254. 29689161 PMC6628205

[ref60] Tremmel P.; Griesser H.; Steiner U. E.; Richert C. How Small Heterocycles Make a Reaction Network of Amino Acids and Nucleotides Efficient in Water. Angew. Chem., Int. Ed. 2019, 58, 13087–13092. 10.1002/anie.201905427. PMC6852251 31276284

[ref61] Sun J.; Vogel J.; Chen L.; Schleper A. L.; Bergner T.; Kuehne A. J. C.; von Delius M. Carbodiimide-Driven Dimerization and Self-Assembly of Artificial, Ribose-Based Amphiphiles. Chem. - Eur. J. 2022, 28, e202104116 10.1002/chem.202104116. 35038189 PMC9303926

[ref62] Dejaegher B.; Heyden Y. Vander. Experimental Designs and Their Recent Advances in Set-up, Data Interpretation, and Analytical Applications. J. Pharm. Biomed. Anal. 2011, 56, 141–158. 10.1016/j.jpba.2011.04.023. 21632194

[ref63] Weissman S. A.; Anderson N. G. Design of Experiments (DoE) and Process Optimization. A Review of Recent Publications. Org. Process Res. Dev. 2015, 19, 1605–1633. 10.1021/op500169m.

[ref64] Aggarwal V. K.; Staubitz A. C.; Owen M. Optimization of the Mizoroki - Heck Reaction Using Design of Experiment (DoE). Org. Process Res. Dev. 2006, 10, 64–69. 10.1021/op058013q.

[ref65] Bowden G. D.; Pichler B. J.; Maurer A. A Design of Experiments (DoE) Approach Accelerates the Optimization of Copper-Mediated F-Fluorination Reactions of Arylstannanes. Sci. Rep. 2019, 9, 11370 10.1038/s41598-019-47846-6. 31388076 PMC6684620

[ref66] When following one axis starting from different corners, it becomes evident that there are different relative improvements of the reaction yields (numbers on the corners) depending on the starting point.

[ref67] The scope does not include arylamines like aniline, which form phosphoramidates that are mostly stable towards hydrolysis.

[ref68] Becker S.; Feldmann J.; Wiedemann S.; Okamura H.; Schneider C.; Iwan K.; Crisp A.; Rossa M.; Amatov T.; Carell T. Unified Prebiotically Plausible Synthesis of Pyrimidine and Purine RNA Ribonucleotides. Science 2019, 366, 76–82. 10.1126/science.aax2747. 31604305

[ref69] Mullen L. B.; Sutherland J. D. Formation of Potentially Prebiotic Amphiphiles by Reaction of β-Hydroxy-n-Alkylamines with Cyclotriphosphate. Angew. Chem., Int. Ed. 2007, 46, 4166–4168. 10.1002/anie.200700394. 17457789

[ref70] Arndt N. T.; Nisbet E. G. Processes on the Young Earth and the Habitats of Early Life. Annu. Rev. Earth Planet. Sci. 2012, 40, 521–549. 10.1146/annurev-earth-042711-105316.

[ref71] Morasch M.; Liu J.; Dirscherl C. F.; Ianeselli A.; Kühnlein A.; Le Vay K.; Schwintek P.; Islam S.; Corpinot M. K.; Scheu B.; Dingwell D. B.; Schwille P.; Mutschler H.; Powner M. W.; Mast C. B.; Braun D. Heated Gas Bubbles Enrich, Crystallize, Dry, Phosphorylate and Encapsulate Prebiotic Molecules. Nat. Chem. 2019, 11, 779–788. 10.1038/s41557-019-0299-5. 31358919

[ref72] Mast C. B.; Schink S.; Gerland U.; Braun D. Escalation of Polymerization in a Thermal Gradient. Proc. Natl. Acad. Sci. U.S.A. 2013, 110, 8030–8035. 10.1073/pnas.1303222110. 23630280 PMC3657786

[ref73] Kudella P. W.; Preißinger K.; Morasch M.; Dirscherl C. F.; Braun D.; Wixforth A.; Westerhausen C. Fission of Lipid-Vesicles by Membrane Phase Transitions in Thermal Convection. Sci. Rep. 2019, 9, 18808 10.1038/s41598-019-55110-0. 31827164 PMC6906453

[ref74] Zhou X.; Chou T.; Aubol B. E.; Park C. J.; Wolfenden R.; Adams J.; Wagner C. R. Kinetic Mechanism of Human Histidine Triad Nucleotide Binding Protein 1. Biochemistry 2013, 52, 3588–3600. 10.1021/bi301616c. 23614568 PMC3835729

