# Supplementary material for: A Ribonucleotide ↔ Phosphoramidate Reaction Network Optimized by Computer-Aided Design
Source: J Am Chem Soc. 2022 Aug 11;144(33):15266–74. doi: 10.1021/jacs.2c05861 (PMC9413217; doi:10.1021/jacs.2c05861)
Supplement: Supplementary file 1 — ja2c05861_si_001.pdf [file ja2c05861_si_001.pdf]

Supporting Information

A Ribonucleotide ↔ Phosphoramidate Reaction Network Optimized by Computer-Aided Design

Andreas Englert[a‡], Julian F. Vogel[a‡], Tim Bergner[b], Jessica Loske[a], Max von Delius[a]*

[a]Institute of Organic Chemistry, Ulm University, Albert-Einstein-Allee 11, 89081 Ulm, Germany

[b]Central Facility for Electron Microscopy, Ulm University, Albert-Einstein-Allee 11, 89081 Ulm, Germany

E-Mail: max.vondelius@uni-ulm.de

Table of Contents

General 3

Abbreviations 4

Synthesis 6

N,N,N,N',N',N',-hexaethyl-2,7-benzenedimethanammonium dibromide 6

Bn-GMP 6

Optimizing Bn-GMP Hydrolysis by DoE 7

Objectives and Responses 7

Input factors 8

Experimental Procedure and Design 9

Data Analysis 10

Additional interaction diagrams regarding the time variable 24

Graphical optimization 14

Optimizing the formation of Bn-GMP by DoE 15

Objectives and responses 15

Input factors 16

Experimental Procedure and Design 17

Data Analysis 20

Graphical Optimization 24

Combining Hydrolysis and Formation of Bn-GMP and Closing the Dissipative Reaction Cycle 26

Reaction cycle 26

HPLC Analysis 26

Further experiments 27

Bn-GMP Reaction cycle at Room Temperature 27

Steady-State Experiment 27

Transient Self-Assembly with AMP and Heptylamine 29

Reaction cycle 29

HPLC Analysis 29

Dynamic Light Scattering (DLS) 30

Confocal Laser Scanning Microscopy (CLSM) 31

Transmission Electron Microscopy (TEM) 32

Cryogenic Transmission Electron Microscopy (cryo-TEM) 33

References 34

Supplementary Data 35

General

All chemicals were purchased from Merck Sigma Aldrich, TCI, AcrosOrganics, FisherScientific, VWR or chemPUR and were used without further purifications. In the case of guanosine 5'-monophosphate, the disodium salt hydrate was purchased. The guanosine 5'-monophosphate content (without crystal water) was determined by quantitative NMR (with internal standard) to be 80%. Since EDC is hygroscopic and susceptible to hydrolysis, it was stored in the freezer and its purity was determined regularly by quantitative HPLC and found to be around 99%.

BrukerAvance 400 and 600 NEO spectrometers were used to record NMR spectra (^{1}H: 400 or 600 Hz, ^{13}C: 101 Hz, ^{31}P: 162 or 243 Hz). The respective spectra were referenced to the residual solvent peaks (1H: 4.79 ppm. Chemical shifts (δ) are denoted in ppm and coupling constants (J) in Hz. Peak multiplicities are indicated as s = singlet, d = doublet, t = triplet and m = multiplet.)

Chromatograms and HRMS were recorded on an Agilent 1260 Infinity II system containing the following modules: G7117C 1260 DAD HS, G7116A 1260 MCT, G7167A 1260 Multisampler, G7104C 1260 flexible pump and 6546 LC/QTOF. HILIC was used as separation mode throughout this study. The UPLC column ACQUITY PRM BEHAmide (100 x 2.1 mm, 1.7 µm, 130 Å) was purchased from Waters and used as stationary phase.

Transmission Electron Microscopy (TEM) measurements were performed on a ZEISS EM10 microscope with an acceleration voltage of 120 kV.

Cryo-TEM grids were analyzed on a JEM-2100F (JEOL) at 200 kV and a temperature of -150 °C using a Gatan cryo-holder.

Dynamic light scattering (DLS) was performed on a Nano-Zetasizer (Malvern Instruments) at 25 °C with a 173° backscatter angle at λ = 633 nm.

Confocal laser scanning microscopy (CLSM) images were recorded with a TCS SP8 confocal microscope using the Leica Application Suite X (LASX) with the LIGHTNING wizard.

DesignExpert 13 (version 13.0.10.0), Wolfram Mathematica (version 12.3.0.0), and OriginPro 2021b (version 9.8.5.201) were used for analyzing and displaying data.

Abbreviations

**Abbreviation**	**Name**
ACN	Acetonitrile
AICc	Akaike Information Criterion
AMP	Adenosine-5’-monophosphate
ANOVA	Analysis of Variance
AppA	*P,P*-Di(adenosine-5′) pyrophosphate (see structure below)
BIC	Bayesian Information Criterion
Bn-GMP	((2R,3S,5R)-5-(2-amino-6-oxo-1,6-dihydro-9H-purin-9-yl)-3-hydroxytetrahydrofuran-2-yl)methyl benzylphosphoramidate
C153	Cumarin 153 (see structure below)
CCD	Central Composite Design
CLSM	Confocal Laser Scanning Microscopy
DAD	Diode Array Detector
DLS	Dynamic Light Scattering
DOE	Design Of Experiment
EDC	1-Ethyl-3-(3-dimethylaminopropyl)carbodiimide (see structure below)
EDC-PA	EDC adducts (see structure below)
EDU	1-(3-(dimethylamino)propyl)-3-ethylurea (see structure below)
EIC	Extracted Ion Chromatogram
ESI	Electrospray Ionisation
EtIm	1-Ethylimidazole
FFD	Full Factorial Design
GMP	Guanosine-5’-monophosphate
GppG	*P,P*-Di(guanosine-5′) pyrophosphate (see structure below)
HILIC	Hydrophilic Liquid Interaction Chromatography
HINT1	Histidine Triad Nucleotide Binding Protein 1
HPLC	High Performance Liquid Chromatography
HRMS	High Resolution Mass Spectrometry
ISDT	Internal Standard
MES	4-Morpholino-ethansulfonic acid
MOPS	3-Morpholino-propansulfonic acid
NMR	Nuclear Magnetic Resonance
OFAT	One Factor at a Time
PA	Phosphoramidate
PRESS	Predicted Residual Sum of Squares
TIC	Total Ion Count
UV/VIS	Ultraviolet/Visible
TEM	Transmission Electron Microscopy

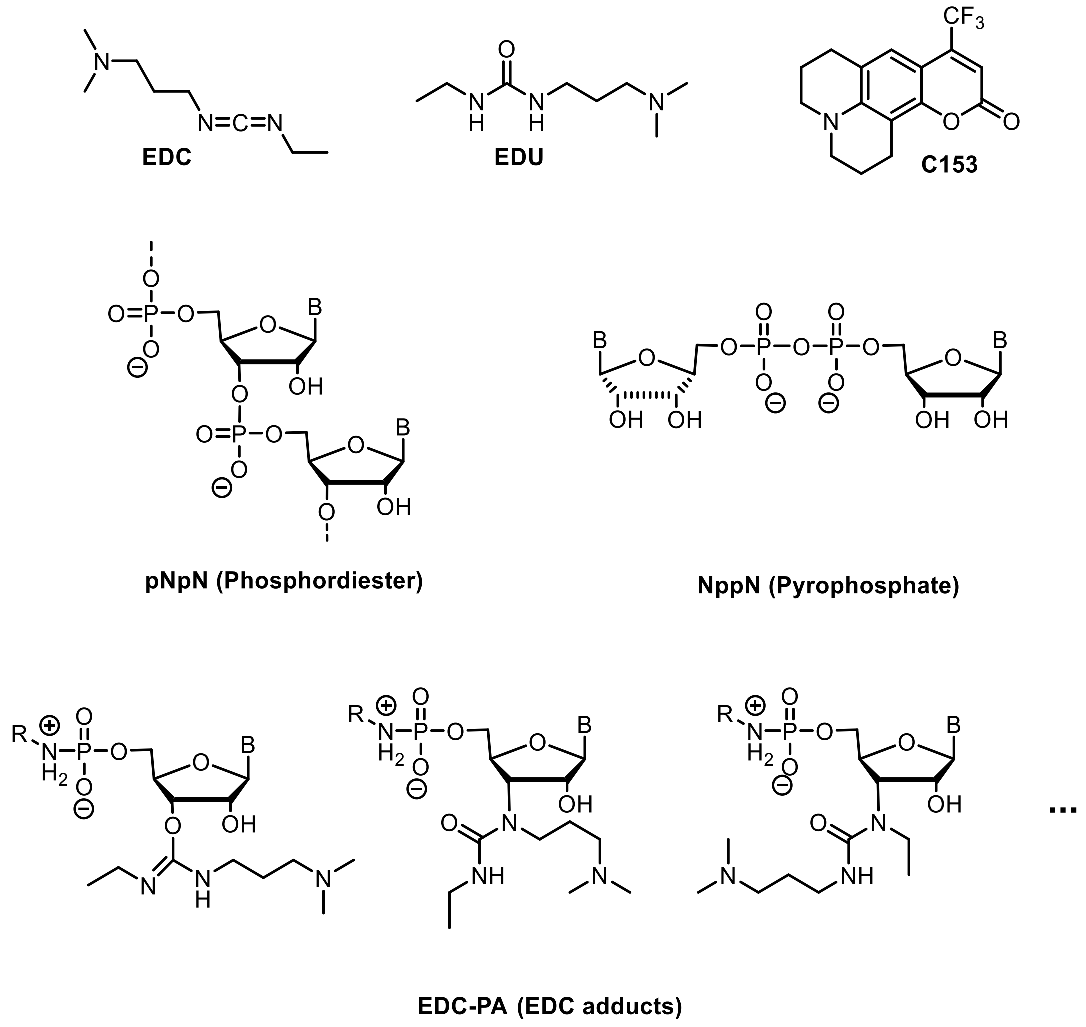

Scheme S1: Full molecular structures and abbreviations for key compounds.

Synthesis

N,N,N,N',N',N',-hexaethyl-2,7-benzenedimethanammonium dibromide

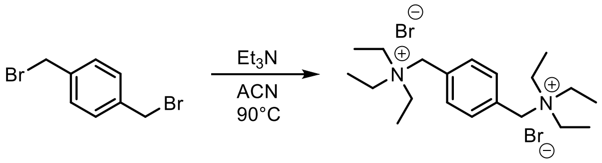

The reaction was performed according to a modified protocol of Chu *et al.*.[1] In a pressure tube 3.00 g (11.4 mmol) 1,4-dibromoxylol were suspended in 15 mL acetonitrile. After addition of 4.75 mL (34.0 mmol, 3 equiv.) triethylamine the mixture was stirred at 90 °C for 24 h. The precipitate was filtered off and washed with cold acetonitrile and diethyl ether. 4.60 g (9.90 mmol 87%) were obtained as a colorless solid.

^{1}H NMR (400 MHz, D_2O, 298 K): δ [ppm] = 7.67 (s, 4 H, phenylic), 4.51 (s, 4 H, benzylic), 3.28 (q,^{3}J= 7.24 Hz, 12 H, CH_2), 1.43 (t, ^{3}J= 7.22 Hz, 18 H, CH_3)

^{13}C NMR (101 MHz, D_2O, 298 K): δ [ppm] = 133.1, 129.5, 59.1, 52.4, 7.0

HRMS (ESI$^+$) (C20H38N2Br1$^+$, calc.: *m/z* = 385.2218): m/z = 385.2216

Bn-GMP

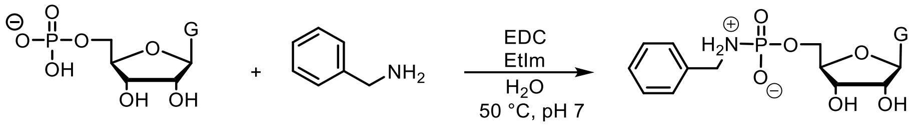

In a 100 mL round bottom flask, 1.43 g guanosine-5'-monophosphate disodium salt (3.50 mmol) were dissolved in 70 mL demineralized water. After addition of 0.46 mL benzylamine (4.20 mmol, 1.2 equiv.), and 1.35 mL 1-ethylimidazole (14.0 mmol, 4.0 equiv.) the reaction mixture was heated to 50 °C. The sample was set to pH = 7 by addition of concentrated HCl. 1.34 g EDC*HCl (7.00 mmol, 2 equiv.) were added and the mixture was stirred at 50 °C for two hours. The reaction mixture was diluted with Acetonitrile (approx. 1:1) and directly injected into a silica-based cartridge (separation mode HILIC, Interchim PF-15HIT-F0025) where the product was eluted by applying a gradient of ACN and NH_4HCO_3 buffer. Combining the respective fractions and lyophilization yielded 1.10 g (2.42 mmol 69%) of Bn-GMP.

^{1}H NMR (400 MHz, D_2O, 298 K): δ [ppm] = 7.97 (s, 1 H, guanosinic), 7.16 (m, 5 H, phenylic), 5.81 (d, 1 H, ^{3}J= 5.71 Hz, 1'), 4.77 (m, 1 H, 2'), 4.36 (m, 1 H, 3'), 4.25 (m, 1 H, 4'), 3.98 (m, 2 H, 5'), 3.75 (m, 2 H, benzylic)

^{13}C NMR (101 MHz, D_2O, 298 K): δ [ppm] = 158.6, 153.6, 140.5 (d), 137.4, 128.1, 127.0, 126.7, 116.3, 86.9, 84.0 (d), 73.0, 70.4, 63.9 (d), 44.9

^{31}P NMR (162 MHz, D_2O, 298 K): δ [ppm] = 8.85

HRMS (ESI$^+$) (C17H22N6O7P1$^+$, calc.: *m/z* = 453.1288): m/z = 453.1285

Optimizing Bn-GMP Hydrolysis by DoE

Objectives and Responses

The hydrolysis of Bn-GMP was investigated, focusing on revealing general trends and optimizing reaction conditions. More specifically, the overall goal of the DOE was to achieve nearly complete hydrolysis (Bn-GMP content < 10-15%) within 24 hours.

Based on this objective the response of interest was chosen to be the relative amount of unhydrolyzed Bn-GMP. The amount was determined by HPLC-MS and *N,N,N,N',N',N'*,-hexaethyl-2,7-benzenedimethanammonium dibromide was used as internal standard. Here, a calibration was conducted where different concentration ratios of the internal standard and Bn-GMP were plotted against the respective intensity ratios. The calibration and the respective chromatograms are depicted in **Figure S1 and Table S1**.

Table S1: Internal Standard Calibration of Bn-GMP.

Run	[Bn-GMP] [mM]	[ISDT] [mM]	[Bn-GMP] / [ISDT]	ISTD intensity	Bn-GMP intensity	R(PA)/R(ISTD)
1	0.1	0.50	0.2	800.87	46.93	0.06
	0.5	0.50	1	824.67	516.08	0.63
	1	0.50	2	803.96	1039.01	1.29
	1.5	0.50	3	792.85	1475.83	1.86
2	0.1	0.50	0.2	799.17	47.89	0.06
	0.5	0.50	1	819.11	517.41	0.63
	1	0.50	2	802.6	1050.13	1.31
	1.5	0.50	3	798.3	1475.6	1.85
3	0.1	0.50	0.2	813.42	45.45	0.06
	0.5	0.50	1	797.54	503.8	0.63
	1	0.50	2	787.42	1031.6	1.31
	1.5	0.50	3	784.49	1458.94	1.86

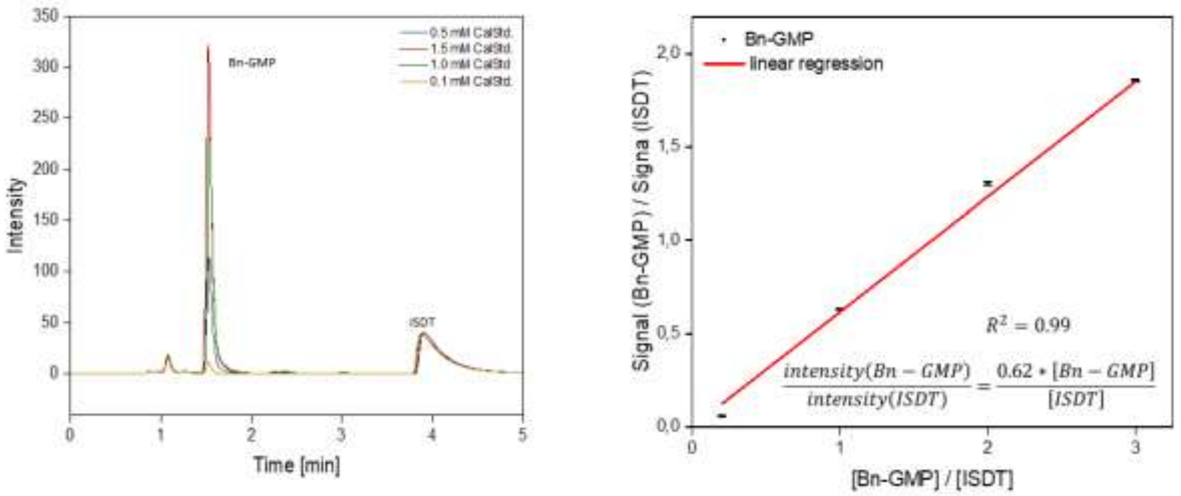

Figure S1: HPLC-based Internal Standard Calibration of Bn-GMP

Input factors

The input factors used in the designed experiment are shown in **Table S2 and Figure S2.**

Table S2: Input factors and ranges used in the designed experiment.

Factor	Name	Units	Type	SubType	Minimum	Maximum	Coded Low	Coded High
A	Temperature	°C	Numeric	Continuous	35.9	64.1	-1 ↔ 40.00	+1 ↔ 60.00
B	pH		Numeric	Continuous	4.6	7.4	-1 ↔ 5.00	+1 ↔ 7.00
C	Time	h	Numeric	Continuous	15.5	32.5	-1 ↔ 18.00	+1 ↔ 30.00
D	Equivalents of EtIm		Numeric	Continuous	0.5	5.93	-1 ↔ 0.50	+1 ↔ 5.00

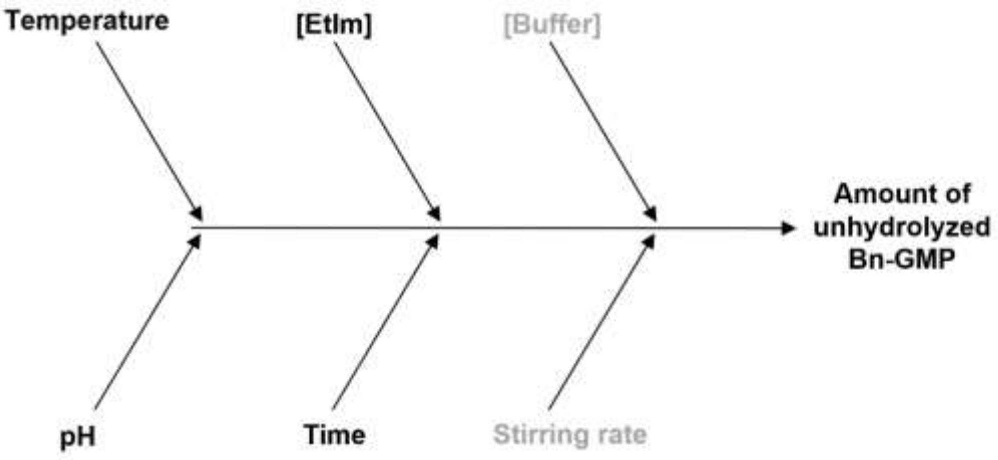

Figure S2: Fishbone diagram of the respective input factors (red), controlled/constant factors (blue) and responses (green).

Experimental Procedure and Design

Stock solutions of MOPS/MES (0.5 M), ISDT (0.05 M), EtIm (1.0 M), and Bn-GMP (0.25 M) were prepared in advance and used during the study. The reactions were performed in a closed reaction vessel equipped with a stirring bar. The reaction mixtures were prepared by pipetting and mixing the desired amounts of stock solutions in the order of buffer, ISDT, demineralized water, EtIm and Bn-GMP. Subsequently the sample was heated, and the pH adjusted by addition of concentrated HCl at the desired temperature. After the respective reaction time, an aliquot was taken from the mixture and analyzed via LC-MS. For HPLC, 50 µL were dissolved in 950 µL MeCN/H_2O (1:1) and 1 µL was injected on a Waters ACQUITY PRM BEHAmide (100 x 2.1 mm, 1.7 µm, 130 Å) column. Eluent: 70% MeCN, 30% aqueous NH_4HCO_3 solution (170 mM, total: 50 mM), flow rate = 0.3 mL/min, temp. = 60 °C.

From previous experiments it was known that the response will not behave in a linear manner based on factor changes. Therefore, an experimental design sufficient of filling a full quadratic description model was used as starting point. In concrete terms this means a CCD design was chosen, where in the first block 20 runs (containing a full factorial design with additional 4 center points) and in the second block 10 runs (containing the axial star points with two additional center points) were conducted as shown in **Table S3**.

Table S3: Experimental Design.

Block	Run	Temperature [°C]	pH	Time [h]	Equivalents of EtIm	Amount of Bn-GMP
Block 1	1	40	7	18	0.5	91
Block 1	2	40	7	18	5	69.9
Block 1	3	50	6	24	2.75	23.48
Block 1	4	60	5	18	5	1.96
Block 1	5	40	5	18	5	35.26
Block 1	6	40	5	30	5	19.31
Block 1	7	50	6	24	2.75	23.71
Block 1	8	60	5	18	0.5	24.8
Block 1	9	40	5	18	0.5	76.23
Block 1	10	60	7	18	0.5	72.26
Block 1	11	50	6	24	2.75	21.8
Block 1	12	60	7	30	0.5	70.77
Block 1	13	60	7	18	5	37.8
Block 1	14	40	7	30	0.5	87.71
Block 1	15	50	6	24	2.75	23.78
Block 1	16	60	5	30	5	1.42
Block 1	17	40	5	30	0.5	63.99

Block 1	18	40	7	30	5	55.43
Block 1	19	60	7	30	5	23.33
Block 1	20	60	5	30	0.5	10.69
Block 2	21	50	6	15.5	2.75	39.53
Block 2	22	50	6	32.5	2.75	16.26
Block 2	23	50	6	24	2.75	18.29
Block 2	24	50	4.6	24	2.75	7.87
Block 2	25	64.1	6	24	2.75	5.73
Block 2	26	50	6	24	0.5	68.38
Block 2	27	50	7.4	24	2.75	74.53
Block 2	28	50	6	24	5.93	6.05
Block 2	29	35.9	6	24	2.75	62.13
Block 2	30	50	6	24	2.75	23.97

Data Analysis

Since the system of interest represents a chemical reaction, the response is limited between boundaries of 0 and 100%. For this reason, the data were at first transformed via a logit function. Based on this, the description model was chosen by applying an AICc model reduction algorithm of all not aliased terms available. **Table S4** shows the respective model and the analysis of variance.

Table S4: ANOVA of the considered description model.

Source	Sum of Squares	df	Mean Square	F-value	p-value	
Block	1.59	1	1.59			
**Model**	74.47	11	6.77	144.98	< 0.0001	significant
A-Temperature	21.91	1	21.91	469.18	< 0.0001	
B-pH	24.57	1	24.57	526.18	< 0.0001	
C-Time	1.93	1	1.93	41.29	< 0.0001	
D-Equivalents of 1-Ethylimidazole	6.71	1	6.71	143.66	< 0.0001	
AB	2.18	1	2.18	46.62	< 0.0001	
AD	0.1321	1	0.1321	2.83	0.1108	
BD	0.2378	1	0.2378	5.09	0.0375	
A^2	0.0284	1	0.0284	0.6082	0.4462	
B^2	0.7301	1	0.7301	15.64	0.001	
D^2	0.9806	1	0.9806	21	0.0003	
A^2D	0.9759	1	0.9759	20.9	0.0003	

**Residual**	0.7938	17	0.0467			
Lack of Fit	0.7266	13	0.0559	3.33	0.1276	not significant
Pure Error	0.0672	4	0.0168			
**Cor Total**	76.85	29				

The regression model and its individual terms (factor effects and interactions) except A^2 and AD are statistically significant and exhibit large F and low p-values. This indicates that the variation explained by the model and its individual terms are large compared to the unexplained variance of the Residual. The model terms A^2 and AD were kept in the model for hierarchy reasons. The high F and low p value of the lack-of-fit test imply that the Lack of Fit is not significant. Further Model statistics are shown in **Table S5**. The predicted coefficient of determination (Predicted R^2) is close to 1 which is caused by a low predicted residual sum of squares (PRESS). This means that the variation explained by the model in "new data" is high, indicating that the model is suited for making predictions. The same holds true for the adjusted coefficient of determination (Adjusted R^2) which measures the variation around the mean explained by the regression taking a penalty for an increasing number of model parameters into account. The difference between Adjusted R^2 and Predicted R^2 is less than 0.2, and therefore in reasonable agreement. The adequate precision can be regarded as a signal-to-noise ratio, where the predicted values of the design points are compared to the average prediction error. As a rule of thumb, a ratio greater than four is desirable which means that the observed ratio of 48.28 indicates an adequate signal.

Table S5: Model Fit Statistics.

Std. Dev.	0.2161	R^2	0.9895
Mean	-0.712	Adjusted R^2	0.9826
C.V. %	30.35	Predicted R^2	0.9568
		Adeq Precision	48.2764

The regression model with the coded factors is depicted in **Table S6**.

Table S6: Coefficients as coded factors of the model regression.

Factor	Coefficient Estimate	df	Standard Error	95% CI Low	95% CI High	VIF
Intercept	-1.2	1	0.0748	-1.36	-1.04	
Block 1	0.034	1				
Block 2	-0.034					
A-Temperature	-1.05	1	0.0483	-1.15	-0.9452	1.00
B-pH	1.11	1	0.0484	1.01	1.21	1.00
C-Time	-0.3104	1	0.0483	-0.4123	-0.2085	1.00
D-Equivalents of EtIm	-1.64	1	0.1366	-1.92	-1.35	7.58
AB	0.3689	1	0.054	0.2549	0.4828	1.00
AD	-0.0909	1	0.054	-0.2048	0.0231	1.00
BD	0.1219	1	0.054	0.0079	0.2359	1.00
A^2	0.0571	1	0.0732	-0.0973	0.2115	1.22
B^2	0.2926	1	0.074	0.1365	0.4486	1.23
D^2	0.4216	1	0.092	0.2275	0.6157	1.62
A^2D	0.6714	1	0.1469	0.3615	0.9812	7.39

Figure S3A-E displays diagnostic graphs of the description model. **Figure S3A** represents the normal Plot of Residuals. Since all data points are following a straight line, it can be assumed that the residuals are normally distributed. Random Fluctuations within the data are not supposed to be systematic and are expected therefore to follow a normal distribution. Potential outliers (not caused by random fluctuations) would have been detected within this plot by strongly deviating from this line. **Figure S3B** shows the predicted and transformed response plotted against their residuals. The data points are randomly scattered, indicating no expanding variance. **Figure S3C** shows the calculated Residuals against the individual run number. The residuals are randomly scattered indicating that no time depending uncontrolled variables are apparent. **Figure S3D** depicts the Residuals within each block. Both blocks exhibit similar ranges of scattering with no specific pattern apparent. **Figure S3E** shows an intuitive way of looking at the performance of the description model in terms of a comparison of predicted and observed response values. A major part of the data variation is explained by the description model. The data scatters around the first bisector. This means that observed responses are in good agreement with the predicted ones. **Figure S3F** represents the Cook's distance and is an example of an influence graph. In this plot, the difference in the predictions of the respective data with and without the run under consideration is calculated, squared, and put in proportion to the estimated variance and the model constant. Thus, the Cook's distance directly shows how much a measured value or potential outlier influences the whole model. Since all runs were below the threshold and no data point stood out in the normal plot either, no further runs were considered as outliers.

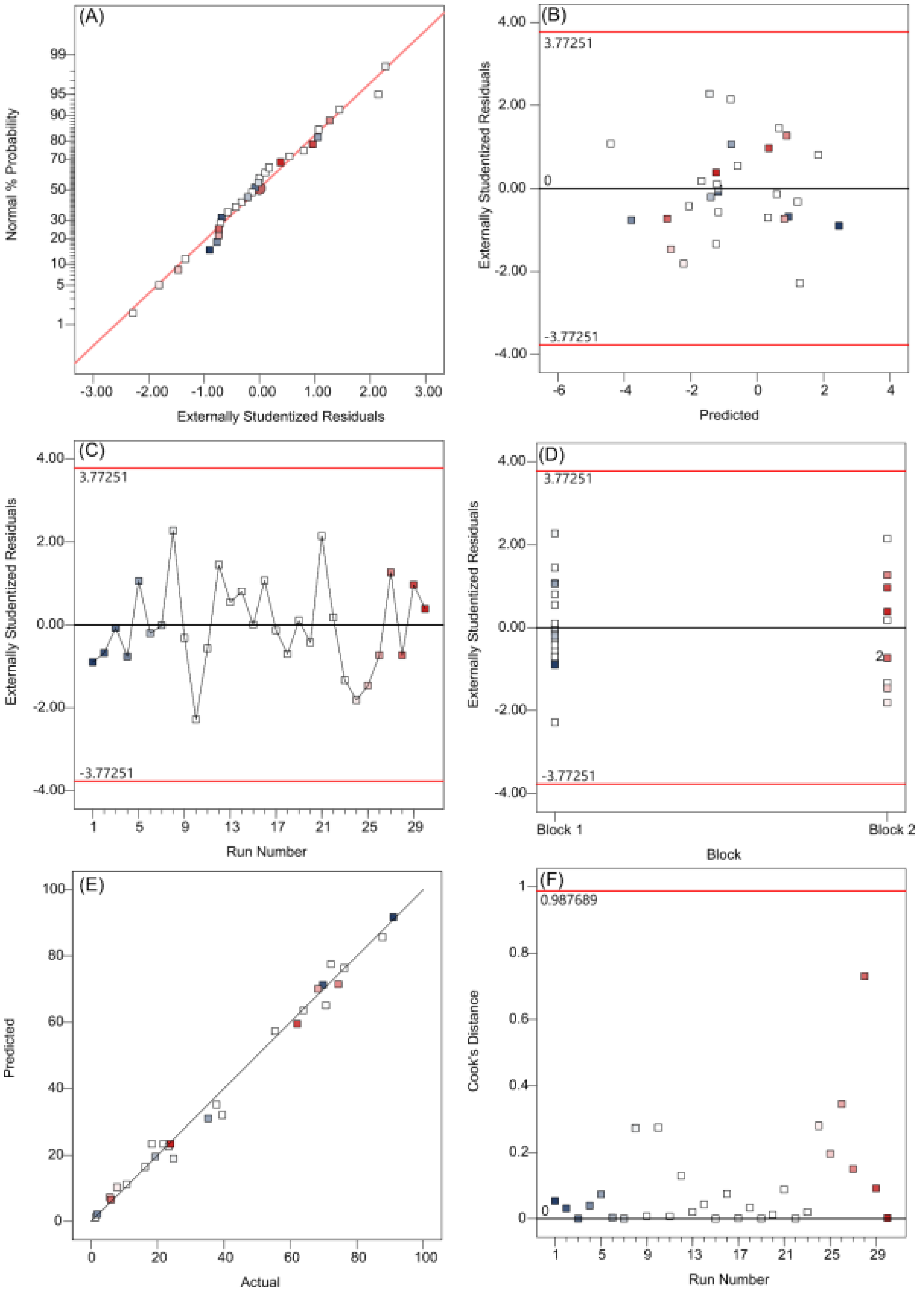

Figure S3: Diagnostic and influence plots. The color of the data points was chosen in accordance with the run number. (blue = 0, red = 30, white = in between). (A) Normal-Plot of Residuals. (B) Residuals vs. Predicted. (C) Residuals vs Runs. (D) Residuals vs. Block. (E) Predicted vs. Actual. (F) Cook's Distance.

Graphical optimization

Figure S4 shows the graphical optimization process of the designed experiment after 24 h and with 5 equiv. of EtIm. In the yellow area the predicted response is smaller than 15%. The adjacent brown area indicates where the 95% confidence interval is below 15%. On this basis, three Confirmation runs (C1, C2 and C3) were conducted (see Figure 2C in the manuscript).

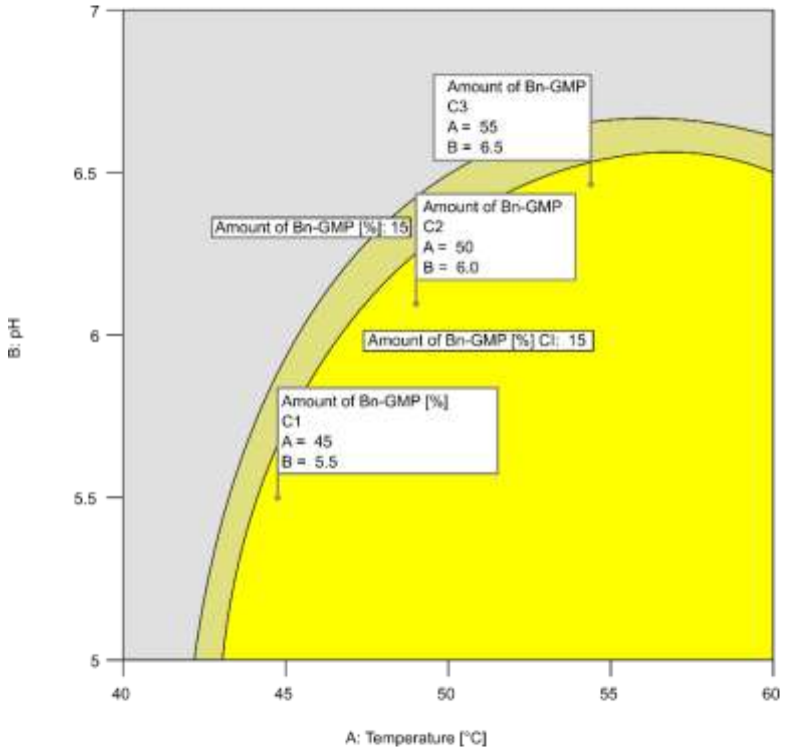

Figure S4: Graphical Optimization of Bn-GMP hydrolysis.

Optimizing the formation of Bn-GMP by DoE

Objectives and responses

The second DoE dealt with the formation of Bn-GMP and the optimization of the forward reaction. In concrete terms this means that the objective was to evaluate if this reaction is suitable to obtain more than at least 50% Bn-GMP.

Based on this objective, the response of interest was chosen to be the relative amount of Bn-GMP formed. RNA and DNA quantification is commonly performed at 260 nm (e.g. OD260 measurements).[2] It was assumed that at this wavelength the only significant absorbing moiety within our system is the purine unit of the nucleotides. To verify this assumption, the Bn-GMP amount determined by internal standard calibration of the previous DOE was compared with the relative area percentage of the Bn-GMP peak in the chromatogram. As depicted in **Table S7**, the relative amount of Bn-GMP formed in the previous DOE was nearly identical with the relative area percentage of the Bn-GMP in the chromatogram at 260 nm, corroborating the validity of our assumption.

Table S7: Bn-GMP amount determinations.

Run	Amount of Bn-GMP [%] determined with ISDT	Relative area percent of Bn-GMP [%]
1	91.00	88.39
14	87.71	85.68
29	62.13	62.34
18	55.43	53.29
7	23.71	22.89
20	10.69	11.48

Based on this observation, it was assumed that the amount of Bn-GMP and other species present can be reasonably determined as relative area percentages in the chromatogram at 260 nm. To further prove the validity of this assumption and to exclude that other species (that were potentially not observed in the chromatographic analysis) are present, a test run was performed to determine the relative amount of Bn-GMP and GMP using quantitative ^{31}P NMR (with TMP as an internal standard). The results were compared with the relative area percentages of the respective LC-MS analysis (see **Table S8**). Even when using a different analytical technique to determine the amount of each species, the relative area percentages in the chromatogram are well comparable to the ones determined by this method. The deviations are within the necessary accuracy for analyzing the reaction network. Furthermore, it becomes apparent that the species observed in the ^{31}P spectra are similar to those monitored in the respective chromatograms, indicating that no (potentially new) species were overlooked that could cause an error in the chromatographic analysis.

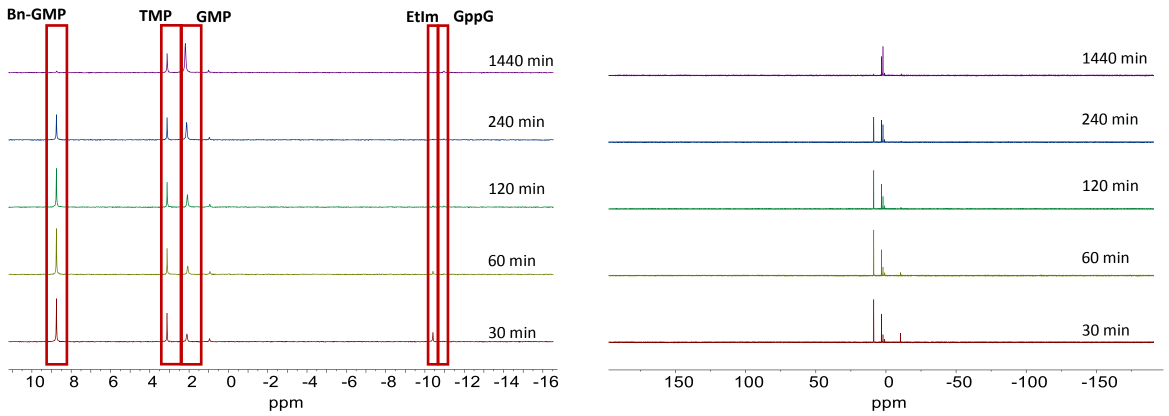

Figure S5: ^{31}P NMR spectra of Bn-GMP.

Table S8: Comparison of the relative amounts of observed species determined by NMR and HPLC.

t [min]	Bn-GMP [%] NMR	Bn-GMP [%] LC	GMP [%] NMR	GMP [%] LC
30	56.67	47.67	22.91	29.32
60	61.51	54.86	24.21	30.50
120	56.11	49.13	33.07	41.09
240	41.48	37.55	50.78	51.02
1440	3.82	3.90	84.95	88.62

Input factors

The input factors used in the designed experiment are shown in **Table S9** and **Figure S6**.

Table S9: Input factors and ranges used in the designed experiment.

Factor	Name	Units	Type	SubType	Minimum	Maximum	Coded Low	Coded High
A	Temperature	°C	Numeric	Continuous	35	65	-1 ↔ 40.00	+1 ↔ 60.00
B	pH		Numeric	Continuous	4.5	7.5	-1 ↔ 5.00	+1 ↔ 7.00
C	Time	min	Numeric	Continuous	30	292	-1 ↔ 30.00	+1 ↔ 240.00
D	Equivalents of EDC		Numeric	Continuous	1.25	7.50	-1 ↔ 1.25	+1 ↔ 6.25
E	[GMP]	mM	Numeric	Continuous	8	48	-1 ↔ 8.00	+1 ↔ 40.00

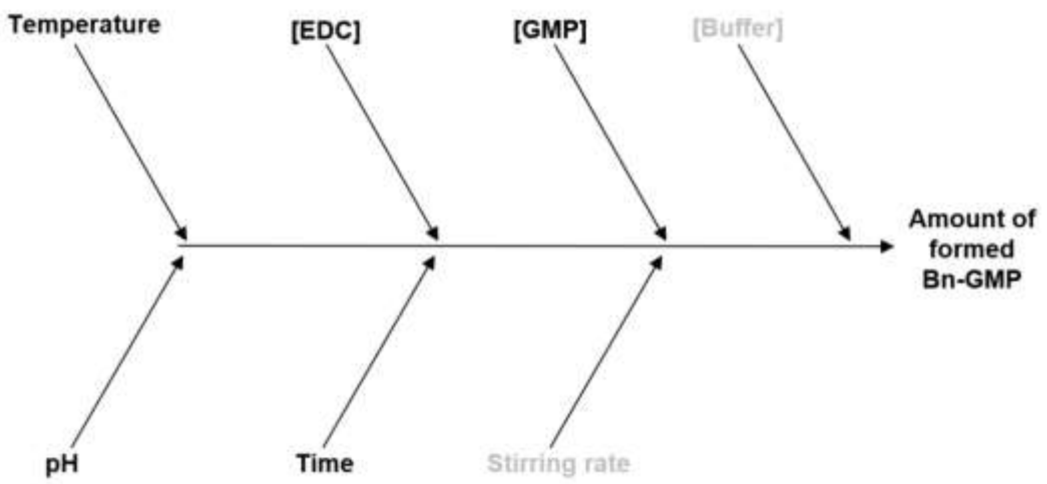

Figure S6: Fishbone diagram of the respective input factors (red), controlled/constant factors (blue) and responses (green).

Experimental Procedure and Design

Stock solutions of MOPS/MES (0.5 M), EtIm (1.0 M), GMP (0.4 M), $BnNH_2$ (1.0 M) were prepared in advance and used during this study. The EDC stock solution (1.25 M) was freshly prepared every day and stored at 7 °C until use. The reactions were performed in HPLC vials containing stirring bars. Reaction mixtures were prepared by pipetting and mixing the desired amounts of stock solutions in the order of buffer, GMP, $BnNH_2$, demineralized water, and EtIm.[3] In all cases, 1.5 equiv. of $BnNH_2$ were used. The sample was heated to the desired temperature and the pH was adjusted with concentrated HCl. The reaction was started by adding the appropriate amount of EDC stock solution to the reaction mixture. For HPLC, 50 µL were dissolved in 950 µL MeCN/H_2O (1:1) and 1 µL was injected on a Waters ACQUITY PRM BEHAmide (100 x 2.1 mm, 1.7 µm, 130 Å) column. Eluent: 70% MeCN, 30% aqueous NH_4HCO_3 solution (170 mM, total: 50 mM), flow rate = 0.3 mL/min, temp. = 60 °C.

From previous experiments it was known that factor changes will not result in a linear change of the response. Therefore, it was assumed that a linear description model would be not sufficient to represent the considered system well. As starting point of the DOE an experimental design was used that is suitable to fill a full quadratic description model. In concrete terms this means a CCD design was chosen, where in the first two blocks 40 runs (containing a full factorial design with additional 8 center points) and the third block 13 runs (containing the axial star points and additional 3 center points) were conducted. Since a full quadratic model was not suited to describe the present system, the experimental design was augmented by optimal exchange methods (Point exchange using an I-optimality criterion). As depicted in **Table S10**, this resulted in an experimental design of 87 runs suitable to fill a full cubic model.

Table S10: Experimental design.

Block	Run	Temperature [°C]	pH	Time [min]	Equivalents of EDC	[GMP] [mM]	Amount of Bn-GMP
Block 1	1	60	5	240	6.25	40	2.55
Block 1	2	60	5	240	1.25	8	1.1
Block 1	3	60	7	30	6.25	40	78.29
Block 1	4	40	7	240	6.25	40	83.21
Block 1	5	40	7	30	6.25	8	26.46
Block 1	6	50	6	135	3.75	24	58.12
Block 1	7	40	5	30	1.25	8	0.18
Block 1	8	40	5	240	1.25	40	2.81
Block 1	9	60	7	240	1.25	40	65.06
Block 1	10	60	5	30	1.25	40	4.12
Block 1	11	40	7	240	1.25	8	34.42
Block 1	12	40	5	30	6.25	40	5.55
Block 1	13	50	6	135	3.75	24	55.07
Block 1	14	60	7	30	1.25	8	32.52
Block 1	15	50	6	135	3.75	24	54.64
Block 1	16	40	5	240	6.25	8	0.87
Block 1	17	40	7	30	1.25	40	36.78
Block 1	18	60	7	240	6.25	8	79.76
Block 1	19	50	6	135	3.75	24	50.94
Block 1	20	60	5	30	6.25	8	1.93
Block 2	21	40	7	30	1.25	8	4.19
Block 2	22	40	5	240	6.25	40	13.67
Block 2	23	60	5	30	1.25	8	0.49
Block 2	24	60	7	240	6.25	40	65.76
Block 2	25	40	7	30	6.25	40	66.59
Block 2	26	50	6	135	3.75	24	61.04
Block 2	27	60	7	30	1.25	40	66.36
Block 2	28	60	7	30	6.25	8	65.03
Block 2	29	60	7	240	1.25	8	49.24
Block 2	30	40	7	240	1.25	40	84.64
Block 2	31	60	5	240	1.25	40	1.11
Block 2	32	60	5	30	6.25	40	14.6
Block 2	33	60	5	240	6.25	8	1.54
Block 2	34	50	6	135	3.75	24	62.15
Block 2	35	40	5	30	1.25	40	1.62
Block 2	36	40	5	240	1.25	8	0.18
Block 2	37	40	5	30	6.25	8	0.52
Block 2	38	40	7	240	6.25	8	73.32
Block 2	39	50	6	135	3.75	24	56.29
Block 2	40	50	6	135	3.75	24	62.44
Block 3	41	50	7.5	135	3.75	24	70.52
Block 3	42	50	6	135	3.75	24	49.36

Block 3	43	50	6	135	3.75	8	29.51
Block 3	44	35	6	135	3.75	24	41.07
Block 3	45	50	6	30	3.75	24	40.31
Block 3	46	50	6	135	3.75	24	61.38
Block 3	47	50	6	292	3.75	24	49.86
Block 3	48	65	6	135	3.75	24	44.58
Block 3	49	50	4.5	135	3.75	24	1.14
Block 3	50	50	6	135	7.5	24	72.1
Block 3	51	50	6	135	3.75	48	72.92
Block 3	52	50	6	135	1.25	24	31.15
Block 3	53	50	6	135	3.75	24	59.69
Block 4	54	40	6.3	240	4.625	40	75.63
Block 4	55	40	6.3	240	2.875	8	31.28
Block 4	56	40	5	170	6.25	29.36	9.34
Block 4	57	53	6.3	30	6.25	8	29.14
Block 4	58	55	6.5	82.5	3.75	32	79.51
Block 4	59	47	5	30	6.25	29.36	7.27
Block 4	60	47	7	170	6.25	8	82.38
Block 4	61	55	5.5	82.5	2.5	32	24.78
Block 4	62	40	7	30	6.25	24	50.48
Block 4	63	47	5	240	1.25	8	0.5
Block 4	64	55	6.5	82.5	3.75	32	84.41
Block 4	65	40	7	240	1.25	24	59.71
Block 4	66	53	7	240	2.875	8	70.72
Block 4	67	55	6.5	82.5	2.5	16	64.86
Block 4	68	40	6.3	100	6.25	40	72.89
Block 4	69	40	7	30	4.625	18.64	35.66
Block 4	70	60	6.3	240	1.25	40	37.38
Block 4	71	45	5.5	135	2.5	32	23.95
Block 4	72	45	6.5	82.5	2.5	32	73.55
Block 4	73	40	7	170	1.25	8	27.12
Block 4	74	55	5.5	187.5	5	16	17.74
Block 4	75	40	7	240	6.25	18.64	90
Block 4	76	40	6.3	240	6.25	29.36	81.49
Block 4	77	40	5	240	2.875	29.36	4.95
Block 4	78	55	6.5	82.5	2.5	16	71.65
Confirmation	79	55	6.1	60	1.88	40	57.13
Confirmation	80	55	6.1	60	1.88	40	53.8
Confirmation	81	55	6.1	60	1.88	40	53.66
Confirmation	82	55	6.1	60	2.75	40	64
Confirmation	83	55	6.1	60	2.75	40	61.6
Confirmation	84	55	6.1	60	2.75	40	61.43
Confirmation	85	55	6.3	60	6.25	40	80.01
Confirmation	86	55	6.3	60	6.25	40	78
Confirmation	87	55	6.3	60	6.25	40	79.07

Data Analysis

Since the forward reaction represents a chemical reaction, the response is limited between the boundaries of 0 and 100%. For this reason, the data was transformed via a logit function. Based on this, the description model was chosen by applying an AICc model reduction algorithm of all not aliased terms available. **Table S11** shows the analysis of variance of the respective model.

Table S11: ANOVA of the considered description model.

Source	Sum of Squares	df	Mean Square	F-value	p-value	
Block	29.38	3	9.79			
**Model**	330.22	22	15.01	213.55	< 0.0001	significant
A-Temperature	2.71	1	2.71	38.49	< 0.0001	
B-pH	37.38	1	37.38	531.81	< 0.0001	
C-Time	3.25	1	3.25	46.19	< 0.0001	
D-EDC	0.0526	1	0.0526	0.7476	0.3912	
E-GMP	26.13	1	26.13	371.74	< 0.0001	
AC	5.65	1	5.65	80.33	< 0.0001	
AD	0.5545	1	0.5545	7.89	0.007	
AE	3.83	1	3.83	54.5	< 0.0001	
BC	3.29	1	3.29	46.77	< 0.0001	
BD	0.0411	1	0.0411	0.5848	0.4479	
BE	1.4	1	1.4	19.87	< 0.0001	
CE	1.81	1	1.81	25.77	< 0.0001	
DE	0.5115	1	0.5115	7.28	0.0094	
A^2	0.9146	1	0.9146	13.01	0.0007	
B^2	10.34	1	10.34	147.15	< 0.0001	
C^2	1.82	1	1.82	25.86	< 0.0001	
D^2	1.15	1	1.15	16.33	0.0002	
E^2	1.2	1	1.2	17.08	0.0001	
ACE	0.974	1	0.974	13.86	0.0005	
BDE	1.17	1	1.17	16.64	0.0002	
B^3	0.9125	1	0.9125	12.98	0.0007	
D^3	0.5411	1	0.5411	7.7	0.0077	
**Residual**	3.66	52	0.0703			
Lack of Fit	3.27	42	0.0778	2.01	0.1189	not significant
Pure Error	0.388	10	0.0388			
**Cor Total**	363.25	77				

The regression model and most of its individual terms (factor effects and higher order interactions) are statistically significant and exhibit large F and low p-values. This indicates that the variation explained by the model as well as its individual terms are quite large compared to the unexplained variance of the Residual. Seemingly insignificant model terms like BD and D were kept within the model for hierarchy reasons. Conversely, the high F and low p-value of the lack-of-fit test imply that the Lack of Fit is not significant. Further Model statistics are shown in **Table S12**. The predicted coefficient of

determination (Predicted R²) is close to 1 which is caused by a low PRESS. This means that the variation explained by the model in "new data" is high, indicating that the model is suited for making predictions. The same holds true for the adjusted coefficient of determination (Adjusted R²) which measures the variation around the mean explained by the regression taking a penalty for an increasing number of model parameters into account. The difference between Adjusted R² and Predicted R² is less than 0.2 meaning that they are in reasonable agreement. The adequate precision can be regarded as a signal-to-noise ratio, where the predicted values of the design points are compared to the average prediction error. As a rule of thumb, a ratio greater than 4 is suited which means that the observed ratio of 578.089 indicates an adequate signal.

Table S12: Model Fit Statistics.

**Std. Dev.**	0.2651	**R^2**	0.9891
**Mean**	-0.8844	**Adjusted R^2**	0.9844
**C.V. %**	29.98	**Predicted R^2**	0.9651
		**Adeq Precision**	57.8089

The regression model with the coded factors is shown in **Table S13**.

Table S13: Coefficients in terms of coded factors of the model regression.

Factor	Coefficient Estimate	df	Standard Error	95% CI Low	95% CI High	VIF
Intercept	0.3233	1	0.0658	0.1913	0.4554	
Block 1	-0.0163	3				
Block 2	0.0144					
Block 3	-0.0289					
Block 4	0.0308					
A-Temperature	0.2442	1	0.0394	0.1652	0.3232	1.1
B-pH	2.53	1	0.1098	2.31	2.75	8.5
C-Time	0.2642	1	0.0389	0.1862	0.3422	1.08
D-EDC	0.1378	1	0.1593	-0.1819	0.4574	17.8
E-GMP	0.766	1	0.0397	0.6862	0.8457	1.05
AC	-0.3766	1	0.042	-0.4609	-0.2923	1.05
AD	-0.1202	1	0.0428	-0.2061	-0.0343	1.07
AE	-0.3201	1	0.0434	-0.4072	-0.2331	1.03
BC	0.2871	1	0.042	0.2029	0.3714	1.03
BD	-0.0321	1	0.042	-0.1163	0.0521	1.03
BE	-0.1971	1	0.0442	-0.2859	-0.1084	1.04
CE	-0.2192	1	0.0432	-0.3058	-0.1326	1.04
DE	-0.1158	1	0.0429	-0.202	-0.0297	1.03
A^2	-0.2585	1	0.0717	-0.4023	-0.1147	1.56
B^2	-0.8627	1	0.0711	-1.01	-0.72	1.55
C^2	-0.4144	1	0.0815	-0.5779	-0.2509	1.78
D^2	-0.3977	1	0.0984	-0.5951	-0.2002	2.66
E^2	-0.3228	1	0.0781	-0.4795	-0.1661	1.68
ACE	-0.1664	1	0.0447	-0.256	-0.0767	1.02

BDE	-0.1831	1	0.0449	-0.2732	-0.0931	1.03
B^3	-0.3408	1	0.0946	-0.5306	-0.151	8.37
D^3	0.4323	1	0.1558	0.1196	0.7449	19.36

Figure S7A-E displays the diagnostic graphs of the model. **Figure S7A** depicts the normal Plot of Residuals. Since all data points are following a straight line, it can be assumed that the residuals are normally distributed. Random Fluctuations within data are not supposed to be systematic and must therefore follow a normal distribution. A potential outlier (not caused by random fluctuations) would have been detected here by deviating from this line. **Figure S7B** shows the predicted transformed response plotted against their residuals. The data points are randomly scattered indicating no expanding variance. **Figure S7C** shows the calculated Residuals against the individual run number. The residuals are randomly scattered indicating that no time depending uncontrolled variables are apparent. **Figure S7D** depicts the Residuals within each block. Both blocks exhibit similar ranges of scattering with no specific pattern apparent. **Figure S7E** depicts an intuitive way of looking at the performance of the description model in terms of a comparison of predicted and observed response values. A great part of the variation of the data is explained by the description model. The data scatters slightly around the first bisector. This means that observed responses are in good agreement with the predicted ones. **Figure S7F** represents Cook's distance and is an example of an influence graph. In this plot, the difference in the predictions of the respective data with and without the run under consideration is calculated, squared, and put in proportion to the estimated variance and the model constant. Thus, the Cook's distance directly shows how much a measured value or potential outlier influences the whole model. Since all runs were below the threshold and no data point stood out in the normal plot either, no further runs were considered as outliers.

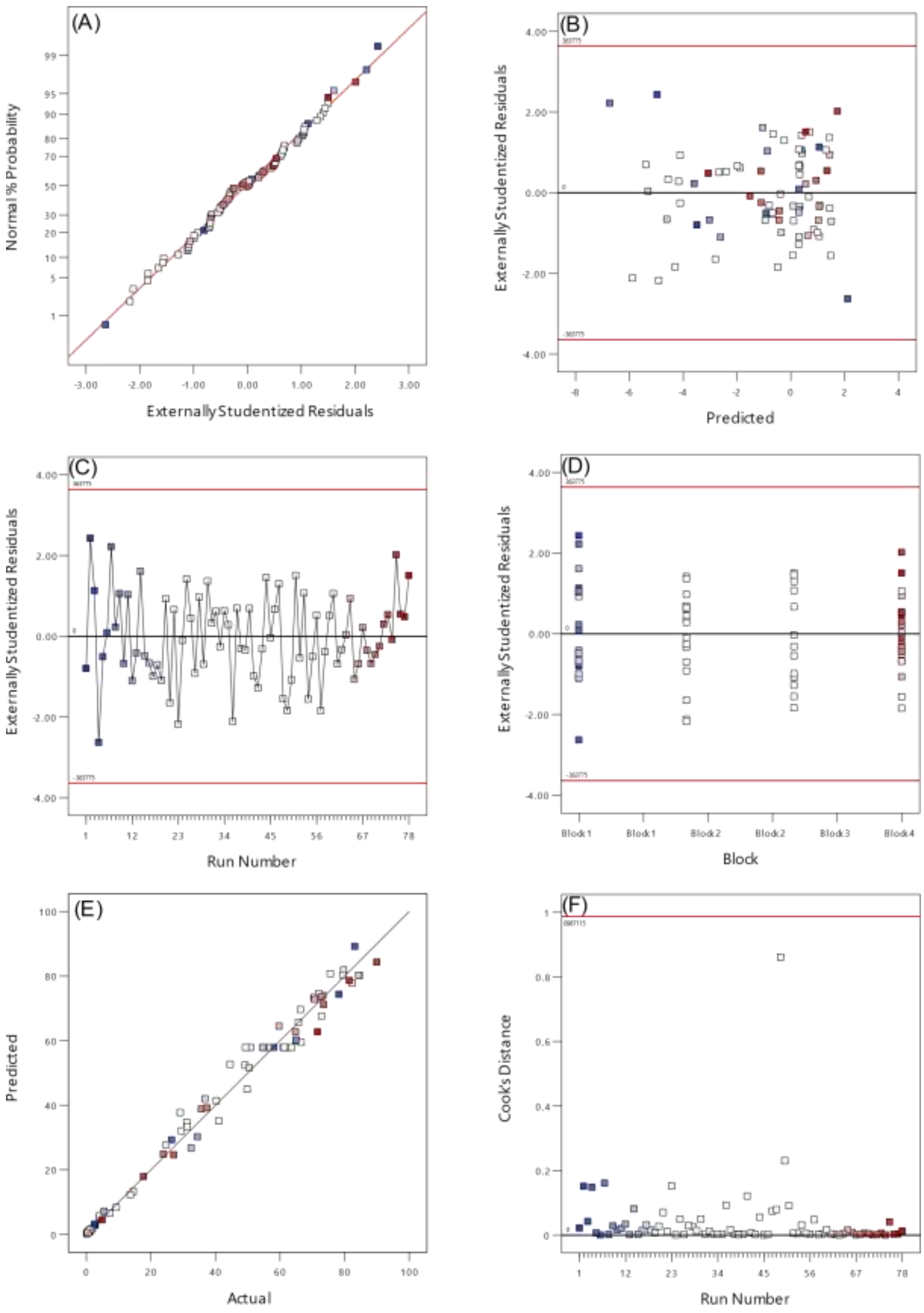

Figure S7: Diagnostic and influence plots. The color of the data points was chosen in accordance with the run number. (blue = 0, red = 78, white = in between). (A) Normal-Plot of Residuals. (B) Residuals vs. Predicted. (C) Residuals vs Runs. (D) Residuals vs. Block. (E) Predicted vs. Actual. (F) Cook's Distance.

Additional interaction diagrams regarding the time variable

The "optimal time" (where the Bn-GMP concentrations is at its peak) varies for every set of parameters. We chose 2.25 h for all diagrams in Fig. 3B; the general trends in the one-factor diagrams, however, do not change significantly when plotted at 1.5 h or 3.5 h instead. The variation of the "optimal time" is especially strong when varying a parameter with strong interactions like the temperature (see interaction diagram in Fig. 3B). Weak but considerable interaction can be found with the parameters [GMP] and pH. In principle, in can be stated that parameter sets that favor hydrolysis (like high temperatures, low pH and high concentration of GMP (where EDC is used up faster)) reach the "optimal time" for the Bn-GMP formation earlier. This can be seen in the two additional interaction diagrams below:

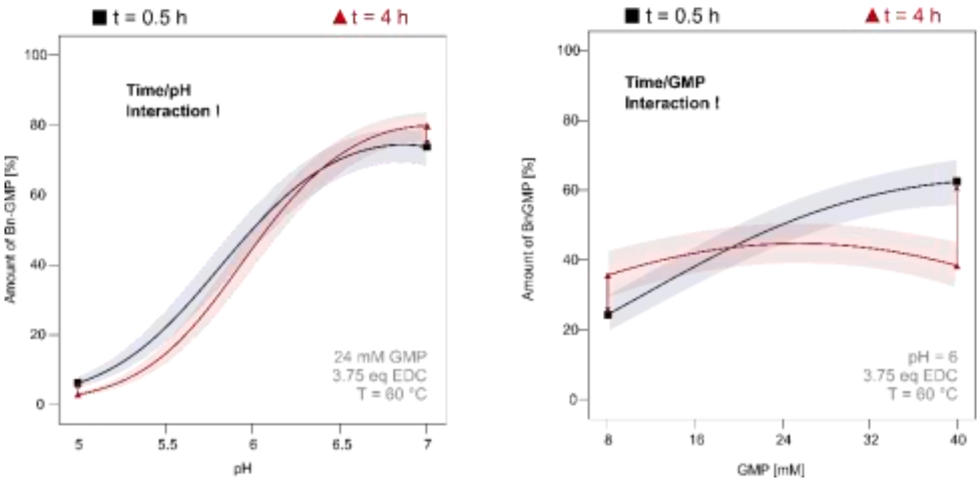

Figure S8: Interaction diagrams of the time/pH and time/[GMP] interaction, respectively.

Graphical Optimization

Figure S9 shows the graphical optimization process of the designed experiment after 60 min, at 55 °C, with 5 equiv. EtIm and with 40 mM GMP. In the yellow area the predicted response (amount of Bn-GMP) is larger than 50%. The adjacent brown area indicates where the 95% confidence interval is larger than 50%. On this basis, three Confirmation runs (C1, C2 and C3) were conducted (see Figure 3C in the manuscript).

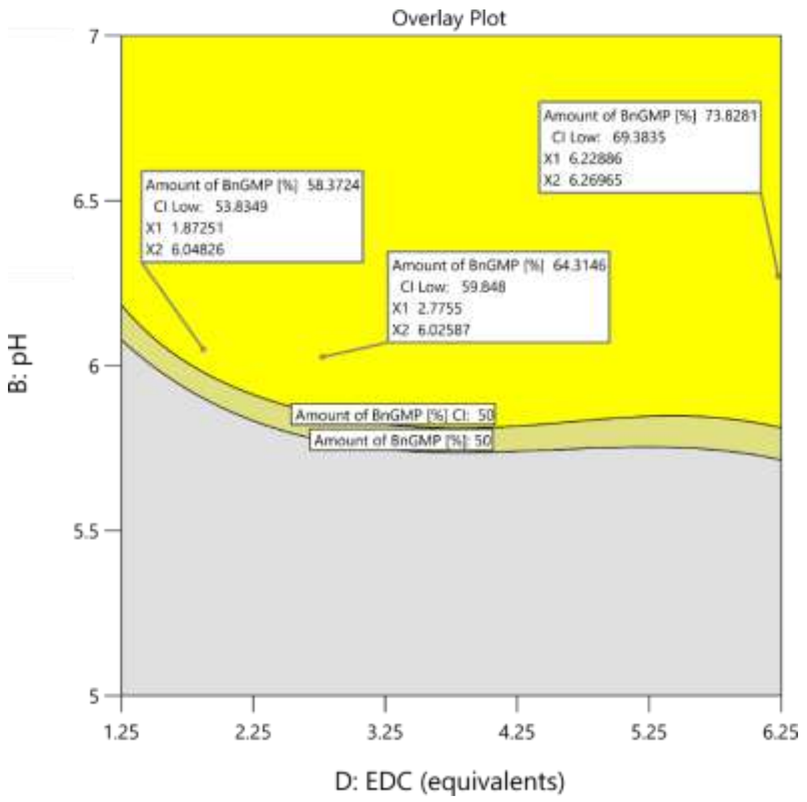

Figure S9: Graphical Optimization and Confirmation of Bn-GMP formation.

Combining Hydrolysis and Formation of Bn-GMP and Closing the Dissipative Reaction Cycle

Reaction cycle

Stock solutions of MOPS/MES (0.5 M), EtIm (1.0 M), GMP (0.4 M), $BnNH_2$ (1.0 M) were prepared in advance and used during this study. To 340 µL water, 100 µL GMP (0.04 mmol, 40 mM, 1 equiv.), 300 µL MOPS/MES buffer (0.15 mmol, 150 mM, 3 equiv.), 60 µL benzylamine (0.06 mmol, 60 mM, 1.5 equiv.) and 200 µL EtIm (0.2 mmol, 200 mM, 5 equiv.) stock solutions were added. The pH was adjusted to pH = 6.1 at 55 °C with conc. hydrochloric acid and 14.4 mg (75.2 µmol, 1.88 equiv.) 1-ethyl-3-(3-dimethylaminopropyl) carbodiimide hydrochloride (EDC) were added at the beginning of each fueled cycle (every 24 h). The reaction mixture was stirred at 55 °C in a tightly closed vial.

HPLC Analysis

The reaction cycle was run according to the procedure described above. For HPLC, 5 µL were dissolved in 995 µL MeCN/H_2O (1:1) and 1 µL was injected on a Waters ACQUITY PRM BEHAmide (100 x 2.1 mm, 1.7 µm, 130 Å) column. Eluent: 70% MeCN, 30% aqueous NH_4HCO_3 solution (170 mM, total: 50 mM), flow rate = 0.3 mL/min, temp. = 60 °C. Compounds were detected at 260 nm with a diode array detector (DAD) and the relative areas of the formed species were used for analysis. Three parallel experiments were performed to obtain error bars as the standard deviation of the three experiments.

Further experiments

Bn-GMP Reaction cycle at Room Temperature

16.3 mg (40 µmol, 40 mM) guanosine 5'-monophosphate (GMP) and 6.5 µL (60 µmol, 60 mM, 1.5 equiv.) benzylamine were dissolved in 1 mL of a freshly prepared aqueous reaction buffer (200 mM 3-morpholinopropionic acid, 200 mM 2-morpholin-4-ylethanesulfonic acid and 200 mM 1-ethylimidazole (5 equiv.)). The pH was adjusted to pH = 6.1 at room temperature with conc. hydrochloric acid. 15.3 mg (80 µmol, 2 equiv.) 1-ethyl-3-(3-dimethylaminopropyl)-carbodiimide hydrochloride (EDC) were added at the beginning to start the reaction. The reaction mixture was stirred at 55 °C in a tightly closed vial.

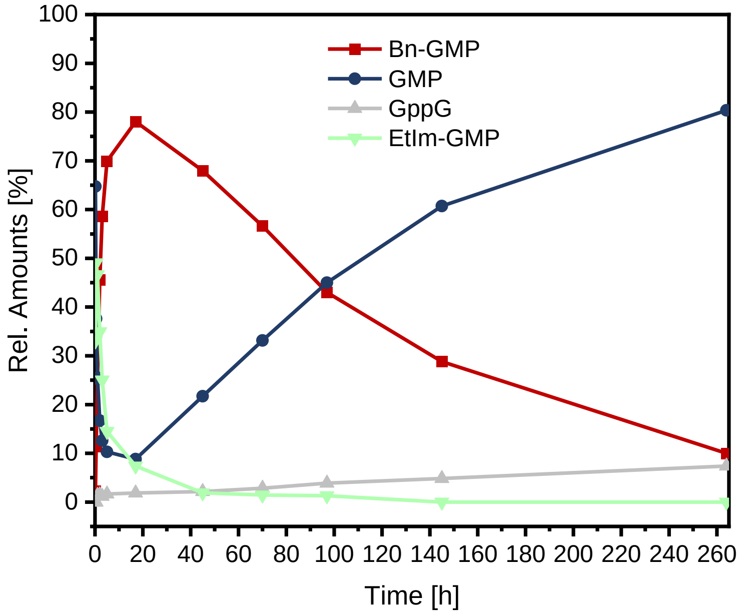

Figure S10: Concentration of GMP, Bn-GMP as well as EtIm-GMP and GppG over 145 h at 25 °C (under otherwise standard conditions).

If the reaction is performed at 25 °C, the formation of Bn-GMP is still rather efficient. The hydrolysis, however, is slowed down significantly with a half-life of the Bn-GMP of around 80 h. This corroborates, that the transient self-assembly could in principle be performed without cycling between two temperatures, albeit with very slow deactivation.

Steady-State Experiment

16.3 mg (40 µmol, 40 mM) guanosine 5'-monophosphate (GMP) and 6.5 µL (60 µmol, 60 mM, 1.5 equiv.) benzylamine were dissolved in 1 mL of a freshly prepared aqueous reaction buffer (200 mM 3-morpholinopropionic acid, 200 mM 2-morpholin-4-ylethanesulfonic acid and 200 mM (5 equiv.) 1-ethylimidazole). The pH was adjusted to pH = 6.1 at 55 °C with conc. hydrochloric acid. With a syringe pump 0.2 equiv. h^{-1} of EDC were added continuously (flowrate = 4 µL h^{-1}, EDC stock = 2 M).

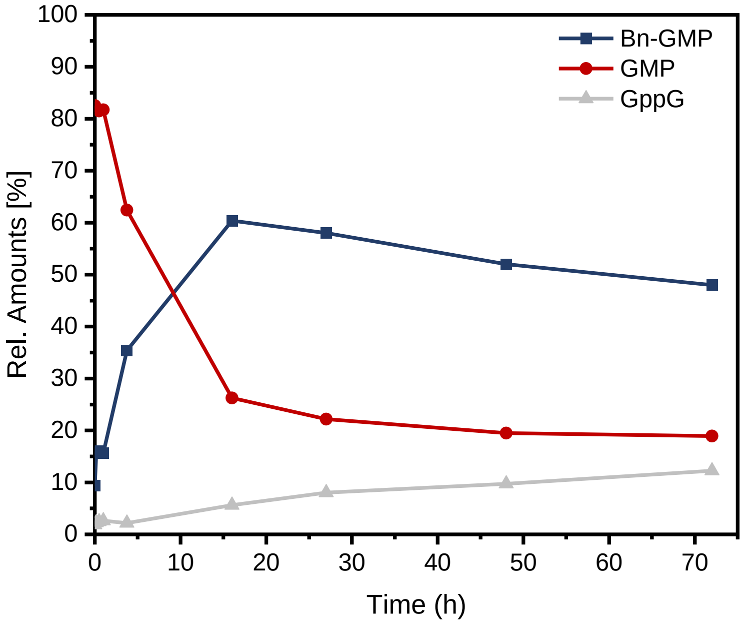

Figure S11: *Concentration of GMP, Bn-GMP and GppG over 72 h under standard conditions with a continuous supply of EDC (0.2 equiv. h^{-1}).*

Continuous addition of the fuel EDC leads to an out-of-equilibrium steady state.

Transient Self-Assembly with AMP and Heptylamine

Reaction cycle

29.4 mg (75 µmol, 75 mM) adenosine 5'-monophosphate (AMP) and 17 µL (0.11 mmol, 113 mM, 1.5 equiv.) heptylamine were dissolved in 1 mL of a freshly prepared aqueous MOPS buffer (200 mM 3-morpholinopropionic acid, 375 mM (5 equiv.) 1-ethylimidazole) and the pH was adjusted to pH = 6.5 at 55 °C with ca. 25 µL conc. hydrochloric acid. 29.0 mg (150 µmol, 2 equiv.) 1-ethyl-3-(3-dimethylaminopropyl)-carbodiimide hydrochloride (EDC) were added at the beginning of each fueled cycle (every 24 h). The reaction mixture was stirred at 55 °C in a tightly closed vial.

HPLC Analysis

The reaction cycle was run according to the procedure described above. For HPLC, 5 µL were dissolved in 995 µL MeCN/H_2O (3:1) and 2 µL were injected on a Waters ACQUITY PRM BEHAmide (100 x 2.1 mm, 1.7 µm, 130 Å) column. Eluent: 75% MeCN, 25% aqueous NH_4HCO_3 solution (170 mM, total: 50 mM), flow rate = 0.3 mL/min, temp. = 60 °C. Compounds were detected at 260 nm with a diode array detector (DAD) and the relative areas of the formed species were used for analysis. Three parallel experiments were performed to obtain error bars as the standard deviation of the three experiments.

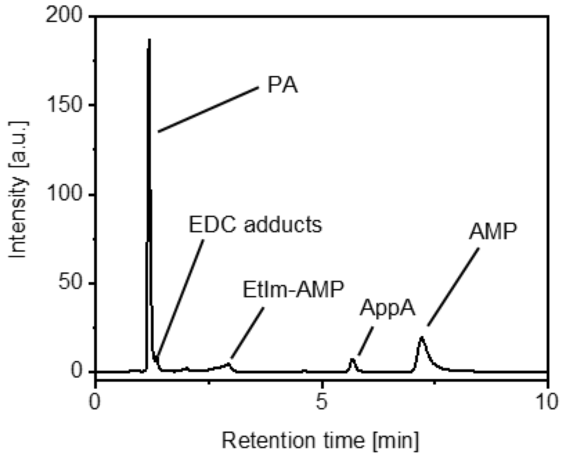

Figure S12: Typical HPLC trace of the reaction cycle with AMP and heptylamine.

Dynamic Light Scattering (DLS)

Samples for DLS were filtered through a 0.45 µm syringe filter before starting the first cycle according to the procedure described above. Dynamic light scattering was performed 1.5 h and 24 h after start of each cycle on a Nano-Zetasizer (Malvern Instruments) at 25 °C with a 173° backscatter angle at λ = 633 nm. At each time point, ten DLS runs were measured. The number mean of five representative measurements each are shown below. The hydrodynamic diameter was obtained as an average of these five measurements while the standard deviations was calculated from the same set of measurements.

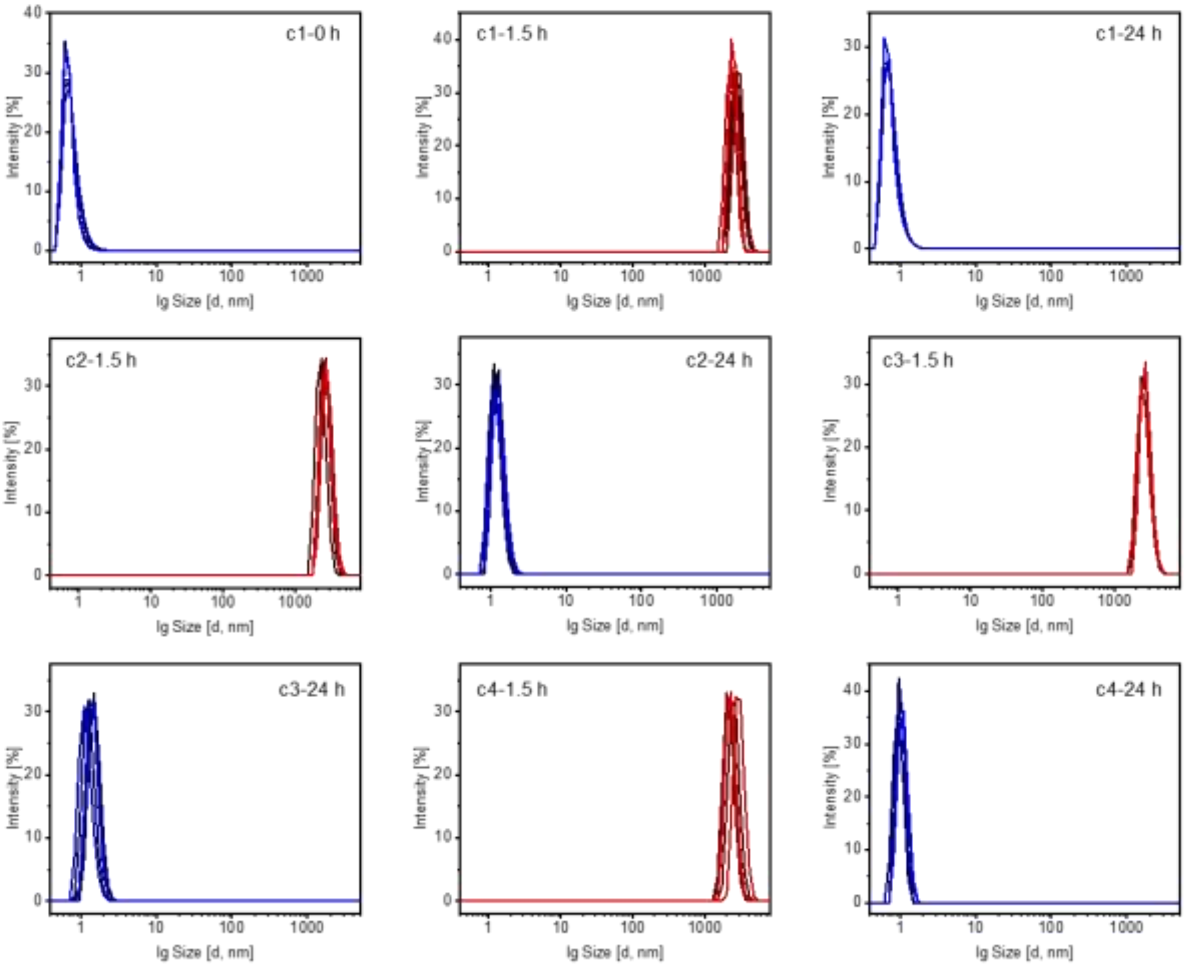

Figure S13: DLS measurements of four dissipative reaction cycles. At each time point five parallel measurements were performed.

Confocal Laser Scanning Microscopy (CLSM)

The reaction cycle was run according to the procedure described above. Before addition of EDC, 1.5 h and 24 h after starting the reaction cycle, samples for CLSM were prepared as follows: 0.5 µL of a 5 mM solution of C153 in THF were added to 1 mL of the sample (final concentration of C153 = 2.5 µM) at 25 °C. A drop of the prepared sample was placed on a freshly cleaned microscope glass slide with a 1 mm well and covered with a cover glass slip. CLSM images were recorded with a TCS SP8 confocal microscope using the Leica Application Suite X (LASX). The fluorescent dye was excited at 405 nm.

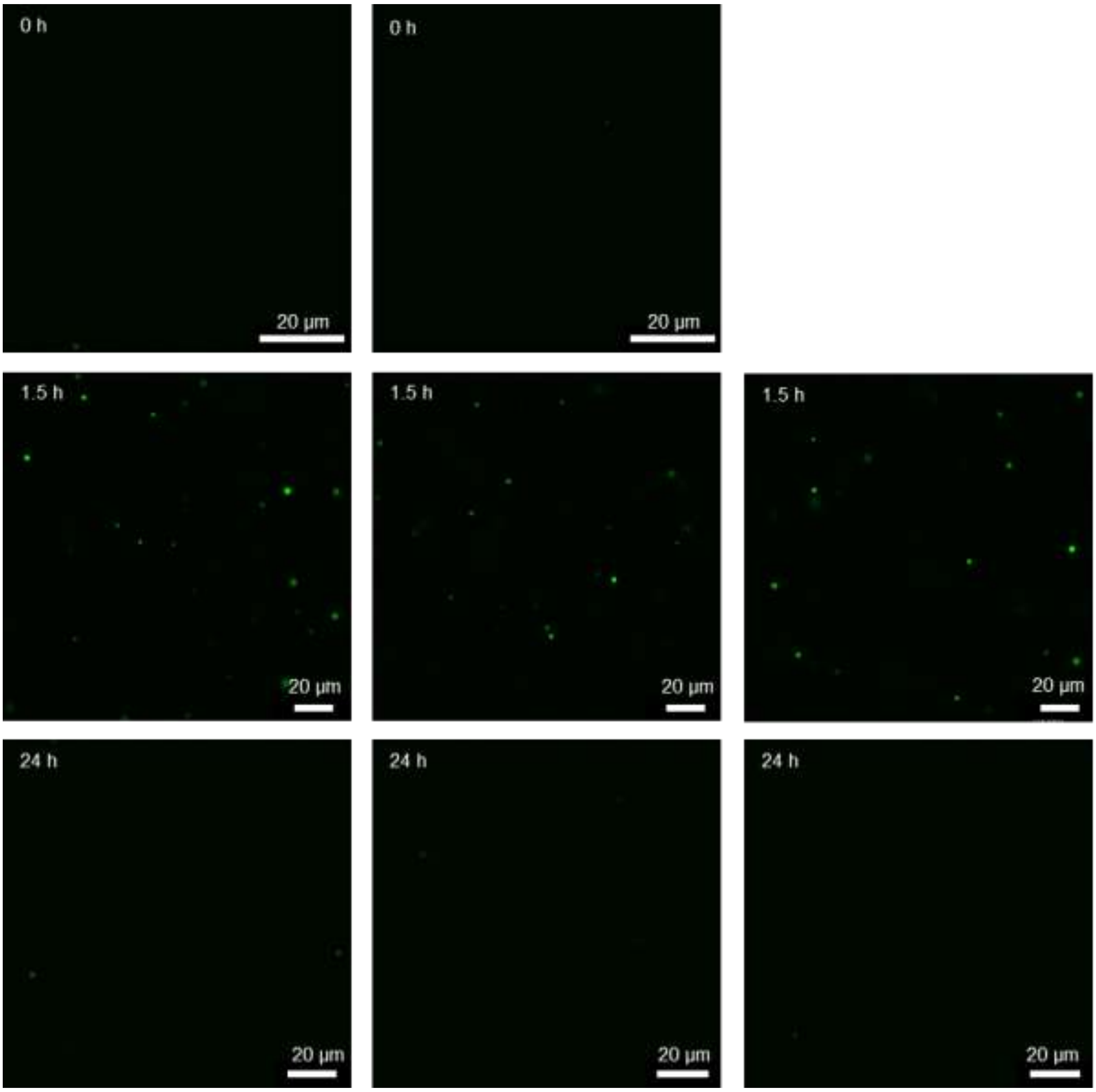

Figure S14: Additional confocal images of the transient self-assembly before EDC addition (top), after 1.5 h (middle) and after 24 h (bottom).

Transmission Electron Microscopy (TEM)

The reaction cycle was run according to the procedure described above. 5 µL of the reaction mixture before (control) and 1.5 h after (aggregated) addition of EDC were deposited on a copper grid at 25 °C. Negative staining with uranyl acetate was performed. TEM measurements were performed on a ZEISS EM10 microscope with an acceleration voltage of 120 kV.

Control experiments showed no aggregates inside the black areas of the stain. After 1.5 h, spherical objects were observed. Their size around 50 – 100 nm differed from the size of the aggregates observed in solution (~ 3 µm), which indicates that they collapse on the surface when being dried.

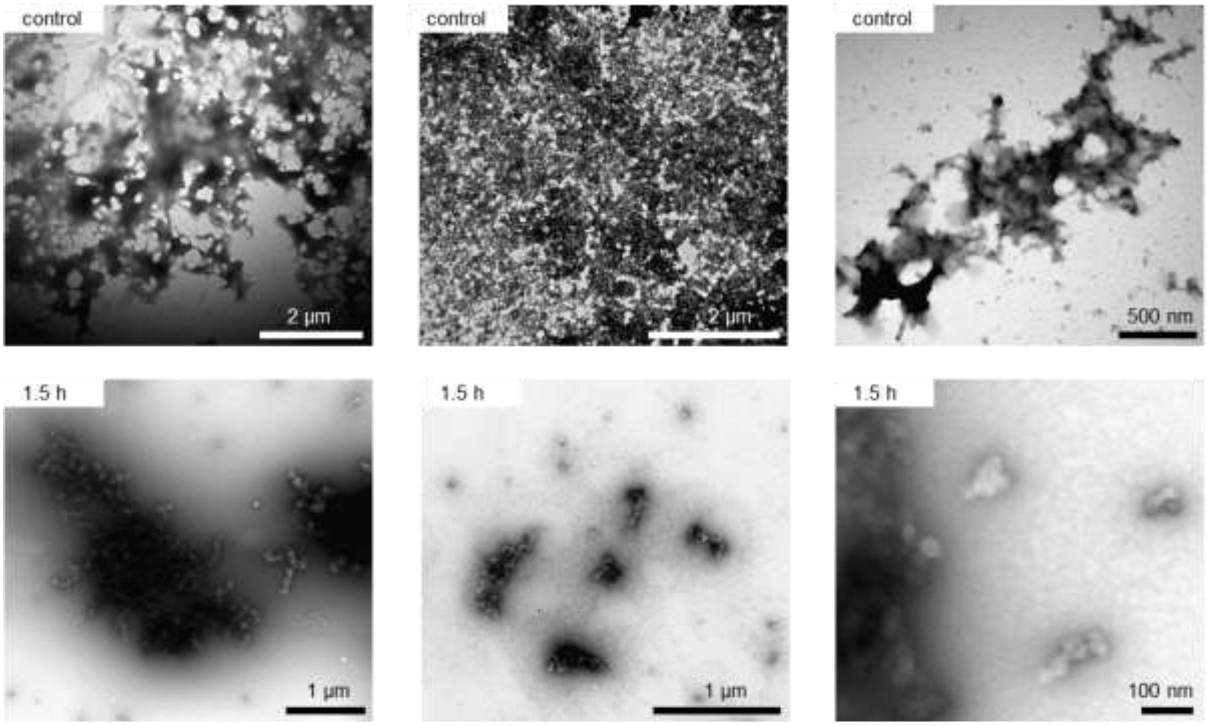

Figure S15: Top: TEM images of the control (before addition of EDC). No aggregates can be observed. Bottom: TEM images 1.5 h after addition of EDC. Clusters of spherical aggregates of about 50 – 100 nm size can be observed.

Cryogenic Transmission Electron Microscopy (cryo-TEM)

The reaction cycle was run according to the procedure described above. 5 µL of the reaction mixture before (control) and 1.5 h after (aggregated) addition of EDC were adhered to a freshly glow-discharged holey carbon grid. After blotting, the grids were vitrified in liquid ethane by a Vitrobot FP 5350/60 (FEI, Eindhoven, Netherlands). The cryo-TEM grids were analyzed on a JEM-2100F (JEOL) at 200 kV and a temperature of -150 °C using a Gatan cryo-holder.

Control experiments showed no aggregates inside the round area of the grid. After 1.5 h, spherical objects were observed. Their size of around one µm differed from the size of the aggregates observed in solution (~ 3 µm), which indicates that they shrink upon freezing. The shape and dark outline is rather indicative for vesicles.

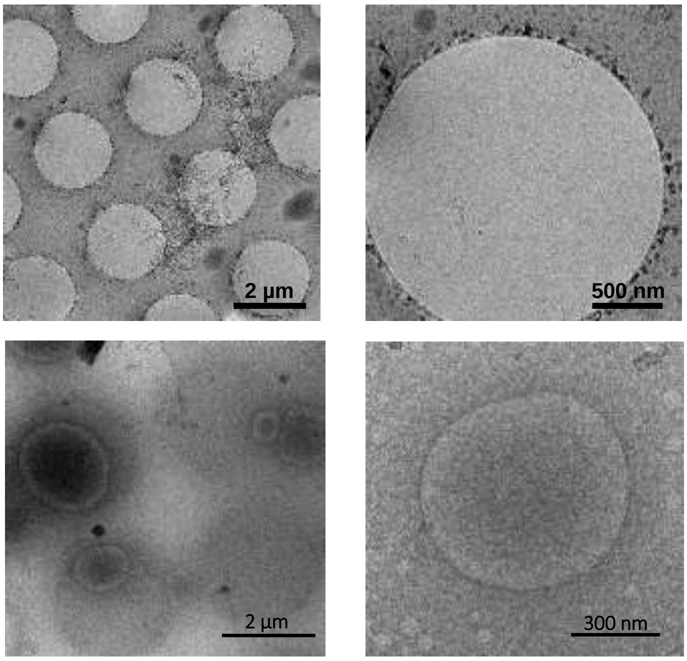

Figure S16: Top: cryo-TEM images of the control (before addition of EDC). No aggregates can be observed. Bottom: cryo-TEM images 1.5 h after addition of EDC. Spherical aggregates of about 200 – 500 nm size can be observed.

References

(1) Fuoss, R. M.; Chu, V. F. H.; Chu, F. H. Conductance of 1,3- 1,4- and 1,5 Di-(Trimethylammonium)-Polymethylene Halides in Water and in Methanol. *J. Am. Chem. Soc.* **1951**, *73*, 949–952.

(2) Barbas, C. F.; Burton, D. R.; Scott, J. K.; Silver, G. L. Quantification of DNA and RNA. *Cold Spring Harb Protoc.* **2007**.

(3) Tremmel, P.; Griesser, H.; Steiner, U. E.; Richert, C. How Small Heterocycles Make a Reaction Network of Amino Acids and Nucleotides Efficient in Water. *Angew. Chem., Int. Ed.* **2019**, *58*, 13087–13092.

Supplementary Data

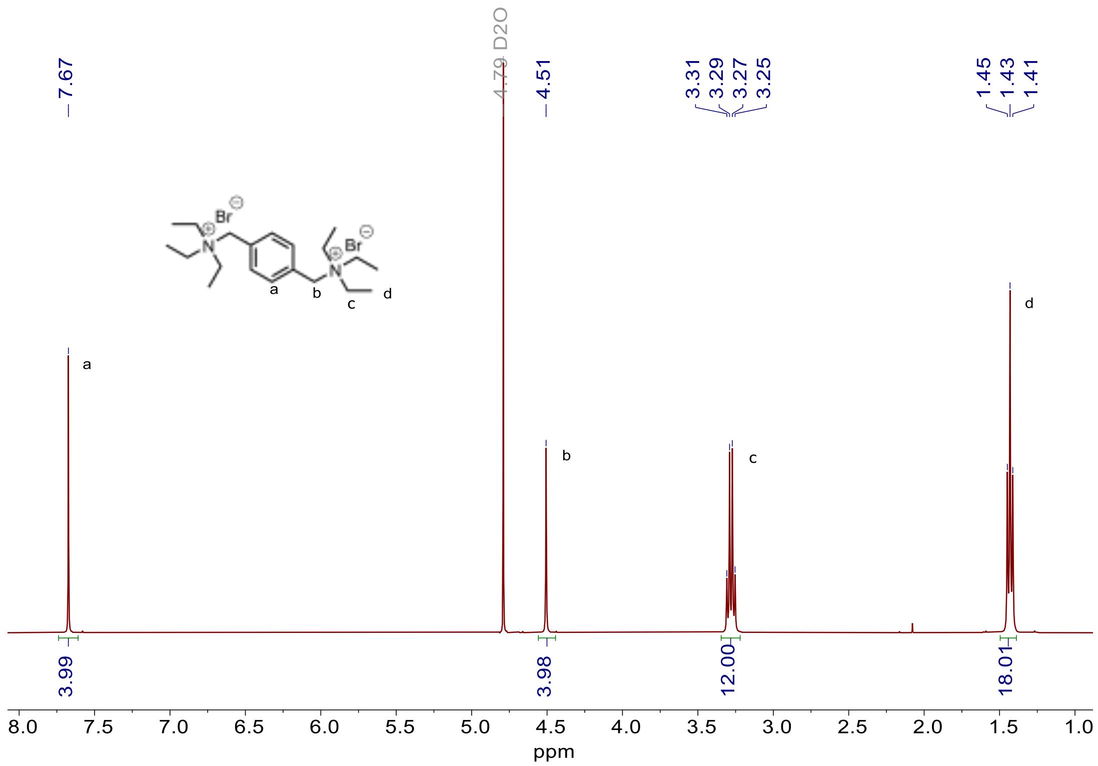

Figure S17: ^{1}H-NMR (400 MHz, D_2O, 298 K) of N,N,N,N',N',N',-hexaethyl-2,7-benzenedimethanammonium dibromide.

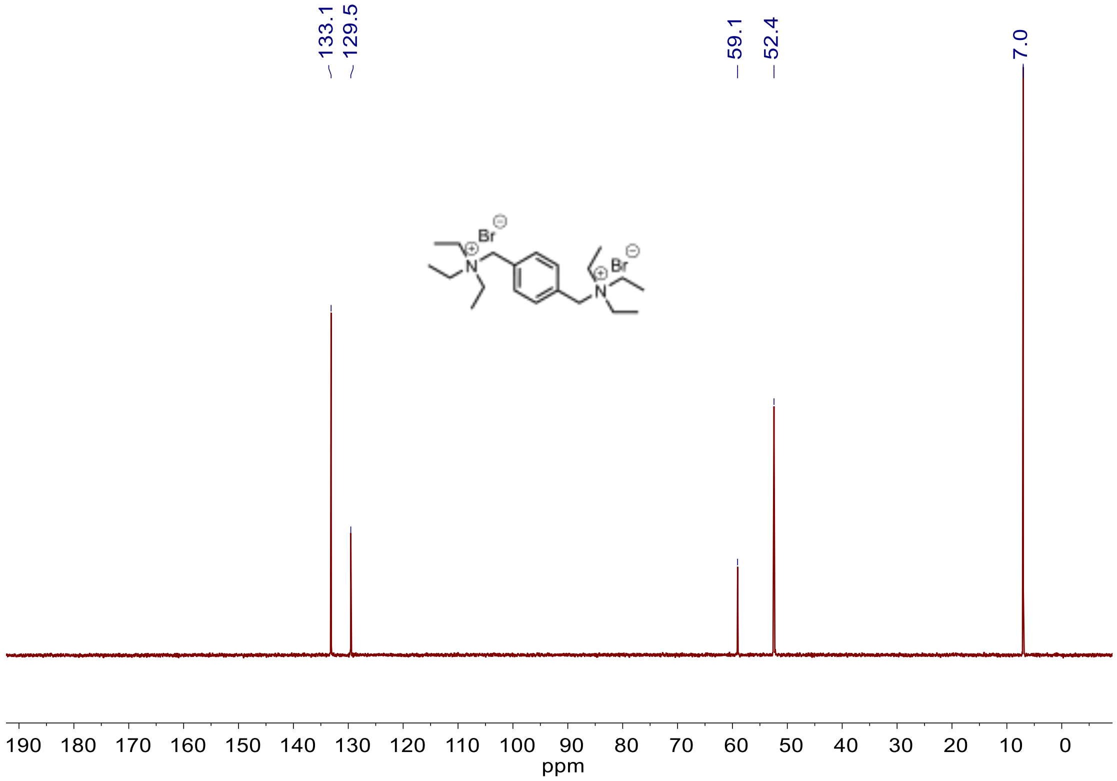

Figure S18: ^{13}C-NMR (101 MHz, D_2O, 298 K) of N,N,N,N',N',N',-hexaethyl-2,7-benzenedimethanammonium dibromide.

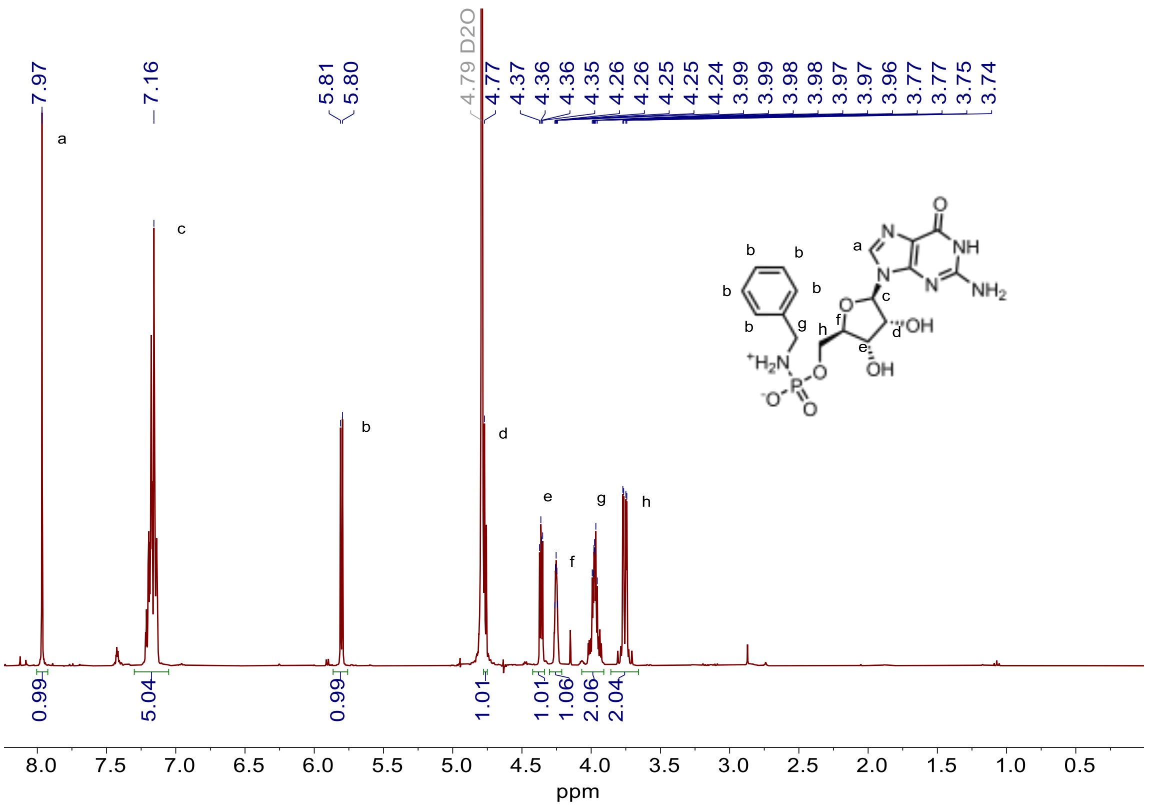

Figure S19: ^{1}H-NMR (400 MHz, D_2O, 298 K) of Bn-GMP.

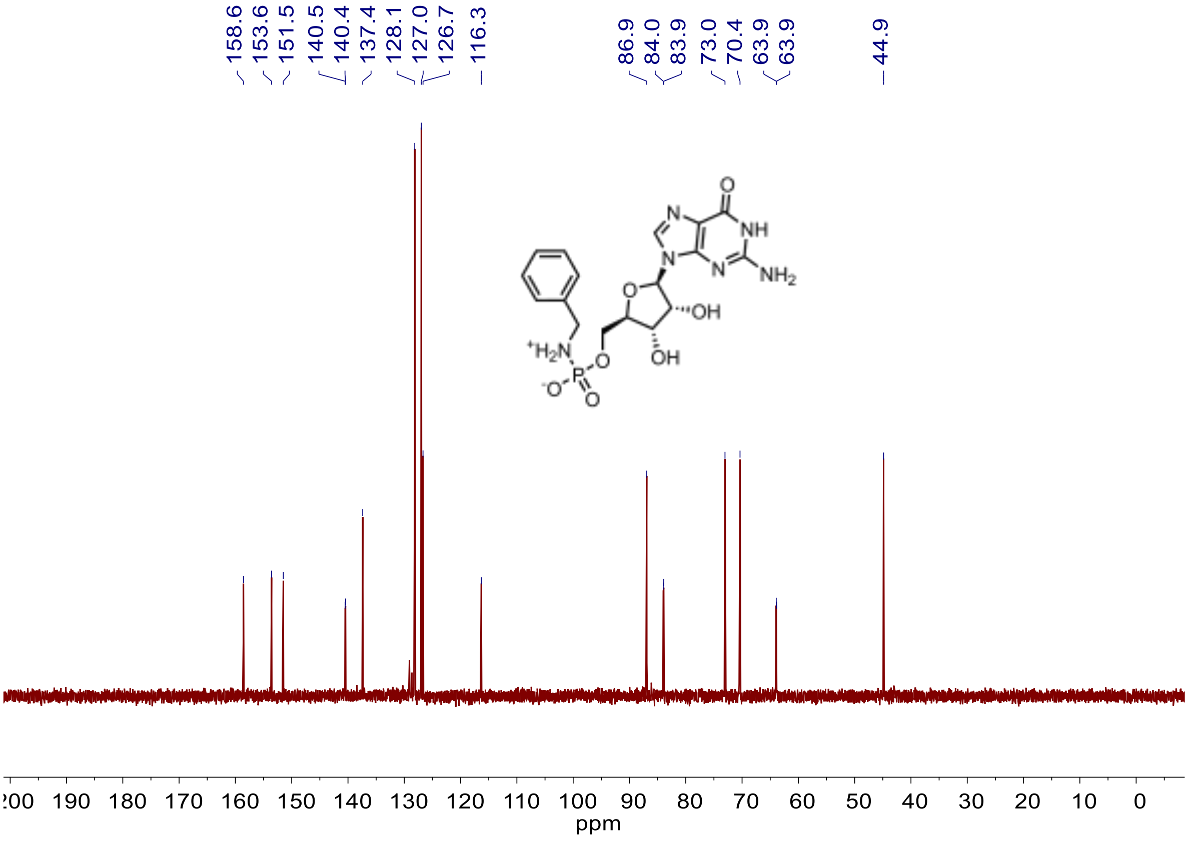

Figure S20: ^{13}C-NMR (101 MHz, D_2O, 298 K) of Bn-GMP.

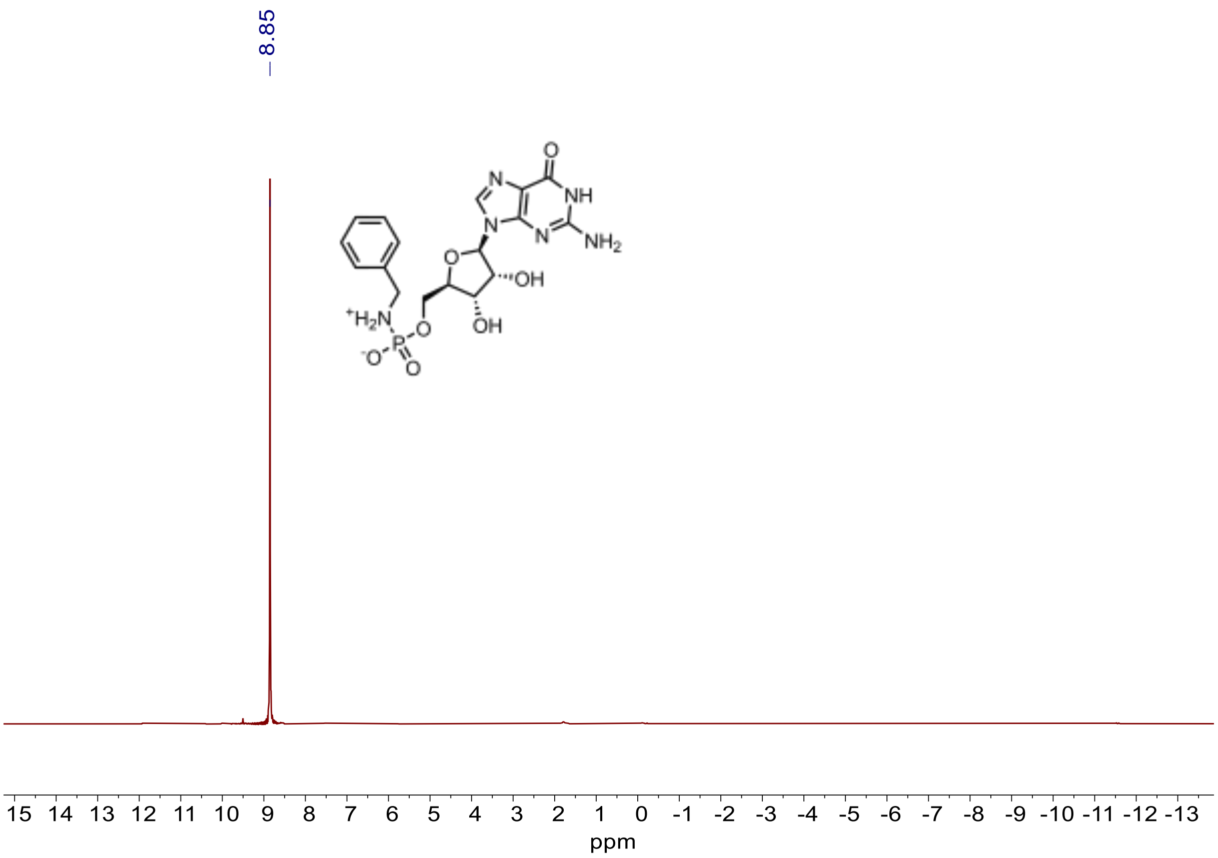

Figure S21: *^{31}P-NMR (162 MHz, D_2O, 298 K) of Bn-GMP.*
